# Molecular basis for diaryldiamine selectivity and competition with tRNA in a type 2 methionyl-tRNA synthetase from a Gram-negative bacterium

**DOI:** 10.1016/j.jbc.2021.100658

**Published:** 2021-04-12

**Authors:** Gustavo Fernando Mercaldi, Maxuel de Oliveira Andrade, Jackeline de Lima Zanella, Artur Torres Cordeiro, Celso Eduardo Benedetti

**Affiliations:** Brazilian Biosciences National Laboratory (LNBio), Brazilian Centre for Research in Energy and Materials (CNPEM), Campinas, SP, Brazil

**Keywords:** *Xanthomonas citri*, MetRS, methionine-tRNA synthetase, diaryldiamines, REP8839, REP3123, Bederocin, antimicrobial resistance, A76, adenosine 76, AaMetRS, *Aquifex aeolicus* MetRS, AaRS, aminoacyl-tRNA synthetase, AMR, antimicrobial resistance, CjIMPDH, *Campylobacter jejuni* Inosine-5'-monophosphate dehydrogenase, CP, connective polypeptide, DMSO, dimethyl sulfoxide, DSF, differential scanning fluorimetry, EcLeuRS, *E. coli* Leucyl-tRNA synthetase, EcMetRS, *Escherichia coli* MetRS, ITC, isothermal titration calorimetry, L-Met, L-methionine, LNBio/CNPEM, Brazilian Bioscience National Laboratory/Brazilian Centre for Research in Energy and Materials, MetRS, methionine-tRNA synthetase, PDB, Protein Data Bank, ScAMD, *Saccharomyces cerevisiae* AMP deaminase, *Tm*, melting temperature, XcΔMetRS, truncated version of XcMetRS lacking the C-terminal extension domain, XcMetRS, *Xanthomonas citri* MetRS

## Abstract

Gram-negative bacteria are responsible for a variety of human, animal, and plant diseases. The spread of multidrug-resistant Gram-negative bacteria poses a challenge to disease control and highlights the need for novel antimicrobials. Owing to their critical role in protein synthesis, aminoacyl-tRNA synthetases, including the methionyl-tRNA synthetases MetRS1 and MetRS2, are attractive drug targets. MetRS1 has long been exploited as a drug target in Gram-positive bacteria and protozoan parasites. However, MetRS1 inhibitors have limited action upon Gram-negative pathogens or on Gram-positive bacteria that produce MetRS2 enzymes. The underlying mechanism by which MetRS2 enzymes are insensitive to MetRS1 inhibitors is presently unknown. Herein, we report the first structures of MetRS2 from a multidrug-resistant Gram-negative bacterium in its ligand-free state and bound to its substrate or MetRS1 inhibitors. The structures reveal the binding mode of two diaryldiamine MetRS1 inhibitors that occupy the amino acid–binding site and a surrounding auxiliary pocket implicated in tRNA acceptor arm binding. The structural features associated with amino acid polymorphisms found in the methionine and auxiliary pockets reveal the molecular basis for diaryldiamine binding and selectivity between MetRS1 and MetRS2 enzymes. Moreover, we show that mutations in key polymorphic residues in the methionine and auxiliary pockets not only altered inhibitor binding affinity but also significantly reduced enzyme function. Our findings thus reinforce the tRNA acceptor arm binding site as a druggable pocket in class I aminoacyl-tRNA synthetases and provide a structural basis for optimization of MetRS2 inhibitors for the development of new antimicrobials against Gram-negative pathogens.

Gram-negative bacteria represent one of the largest groups of prokaryotes, many of which are pathogens that infect a broad range of organisms, from plants to humans. These bacterial pathogens are not only responsible for important losses in agriculture and livestock production but are also the major cause of nosocomial infections worldwide. In fact, one of the major concerns regarding human bacterial infections nowadays is the escalating development of antimicrobial resistance (AMR) developed by many Gram-negative pathogens (1, 2, 3, 4, 5). The costly impacts of AMR on mortality and morbidity across the globe have led the World Health Organization to establish a priority pathogen list along with guidelines for research and development of new antimicrobials (2, 6).

Methionyl-tRNA synthetase (MetRS) is a class I aminoacyl-tRNA synthetase (AaRS) that plays an essential role in genetic code translation. MetRS recognizes both the initiator and elongator cognate tRNAs and charges L-methionine (L-Met) to their acceptor arms (7, 8, 9). This process occurs *via* a two-step reaction that requires ATP to form the intermediate aminoacyl-adenylate (aa-AMP) before L-Met is attached to the tRNA:

aminoacid(aa)+ATP+AaRS→aa-AMP-AaRS+PPi(step1)

aa-AMP-AaRS+tRNA→AaRS+aa-tRNA+AMP(step2)

Two forms of MetRS are found in nature, MetRS1 and MetRS2. Generally, MetRS1 is found in Gram-positive bacteria, protozoan parasites, and mitochondria, whereas MetRS2 is mainly found in Gram-negative bacteria and archaea and in the cytosol of eukaryotic cells (10, 11, 12, 13). Based on the structural organization of the connective polypeptide (CP) domain, MetRS proteins are further classified into four families (14). Thus, MetRS1 has a CP domain with a single knuckle that binds one (family C) or none (family D) zinc ion. On the other hand, the CP domain of MetRS2 has two knuckles that bind two (family A) or one (family B) zinc ion.

Thanks to their critical roles in protein synthesis and to the evolutionary divergence that exists between hosts and pathogens, AaRS enzymes have attracted the attention of many research groups and pharmaceutical companies interested in antimicrobial drug development (15, 16, 17, 18). Mupirocin and tavaborole, for instance, are prominent examples of AaRS inhibitors that are in clinical use to fight skin infections caused by bacteria and fungi, respectively (19, 20, 21, 22).

Like other AaRS, MetRS is regarded as a promising target for the development of antimicrobial agents. Methionine and methionyl-adenylate analogues were among the first MetRS inhibitors to be developed (23, 24, 25, 26). Nevertheless, although many of these molecules were active against bacterial pathogens, they lacked selectivity, which restricted their use to biochemical and structural studies only (27). By contrast, an aminoquinolinone-based inhibitor of *Staphylococcus aureus* MetRS identified by high-throughput screening (28) served as inspiration for the development of potent derivatives with activities over a wide range of protozoans and Gram-positive pathogens (29, 30, 31, 32, 33, 34, 35, 36, 37, 38, 39, 40, 41, 42, 43, 44, 45, 46, 47, 48).

Efforts to develop new MetRS inhibitors have been mostly devoted to MetRS1 enzymes. Importantly, however, pathogens carrying genes coding for MetRS2, like most Gram-negative bacteria, are less sensitive or insensitive to MetRS1 inhibitors (10, 11, 42, 44).

Despite the significant progress made so far to establish the determinants of MetRS1 interaction with inhibitors (32, 34, 35, 36, 37, 38, 48, 49), the precise structural basis of why MetRS2 enzymes are insensitive to MetRS1 inhibitors is still unknown. In addition, the functional relevance of the so-called ‘auxiliary pocket’, usually occupied by MetRS1 inhibitors, remains elusive.

In this work, we report the first free and ligand-bound structures of the single MetRS2 enzyme (XcMetRS) found in the Gram-negative bacterium *Xanthomonas citri*, an economically important citrus disease pathogen (50, 51) that, as shown here, is naturally resistant to multiple antibiotics. The structures reported here revealed the binding mode of two MetRS1 inhibitors (REP8839 and REP3123) that occupy both the methionine site and so-called auxiliary pocket formed upon ligand binding. Our data also provide a structural basis for the selectivity of MetRS1 inhibitors between MetRS1 and MetRS2 enzymes, which is consistent with the observation that such inhibitors display low binding affinities to XcMetRS and weakly inhibited *X. citri* growth. The low affinity of XcMetRS to REP8839, for instance, was reverted by a mutation in the auxiliary pocket, which, as proposed here, is the binding site of the tRNA acceptor arm, a feature that appears to prevail in all class Ia AaRS.

## Results

### *X. citri* as a Gram-negative plant pathogen model for natural antibiotic resistance

*X. citri* is a Gram-negative bacterium that colonizes all commercial citrus varieties (50, 51). Some *X. citri* strains were reported to be resistant against bacteriocins, and penicillin (52). To investigate whether the *X. citri* strain 306 also displays natural resistance against antibiotics, we performed antibiograms using agar disc-diffusion assays. The results reveal that the *X. citri* strain 306 is resistant to several beta-lactams including ampicillin and amoxicillin, monobactams (Aztreonam), cephalosporins of first (cephalothin), second (cefaclor and cefoxitin), third (cefotaxime, ceftriaxone, and ceftazidime), and fourth (cefepime) generations, and to imipenem, which are widely used in clinical medicine (Fig. S1). In addition, *X. citri* is resistant to nitrofurantoin, a nitrofuran antibiotic commonly used to treat urinary tract infections and showed inhibition halos for tobramycin and neomycin that are close to breakpoint values considered for a resistant phenotype (Fig. S1). This makes *X. citri* an attractive Gram-negative plant pathogen model for studies of AMR and susceptibility to new antibiotics. Taking this into account and considering that *X. citri* carries a single MetRS gene, this work provides the first biochemical and structural characterization of a phytopathogen MetRS that can be exploited as a drug target.

### *X. citri* MetRS is an oligomeric type 2 enzyme with high affinity for L-Met

**Figure 1:**
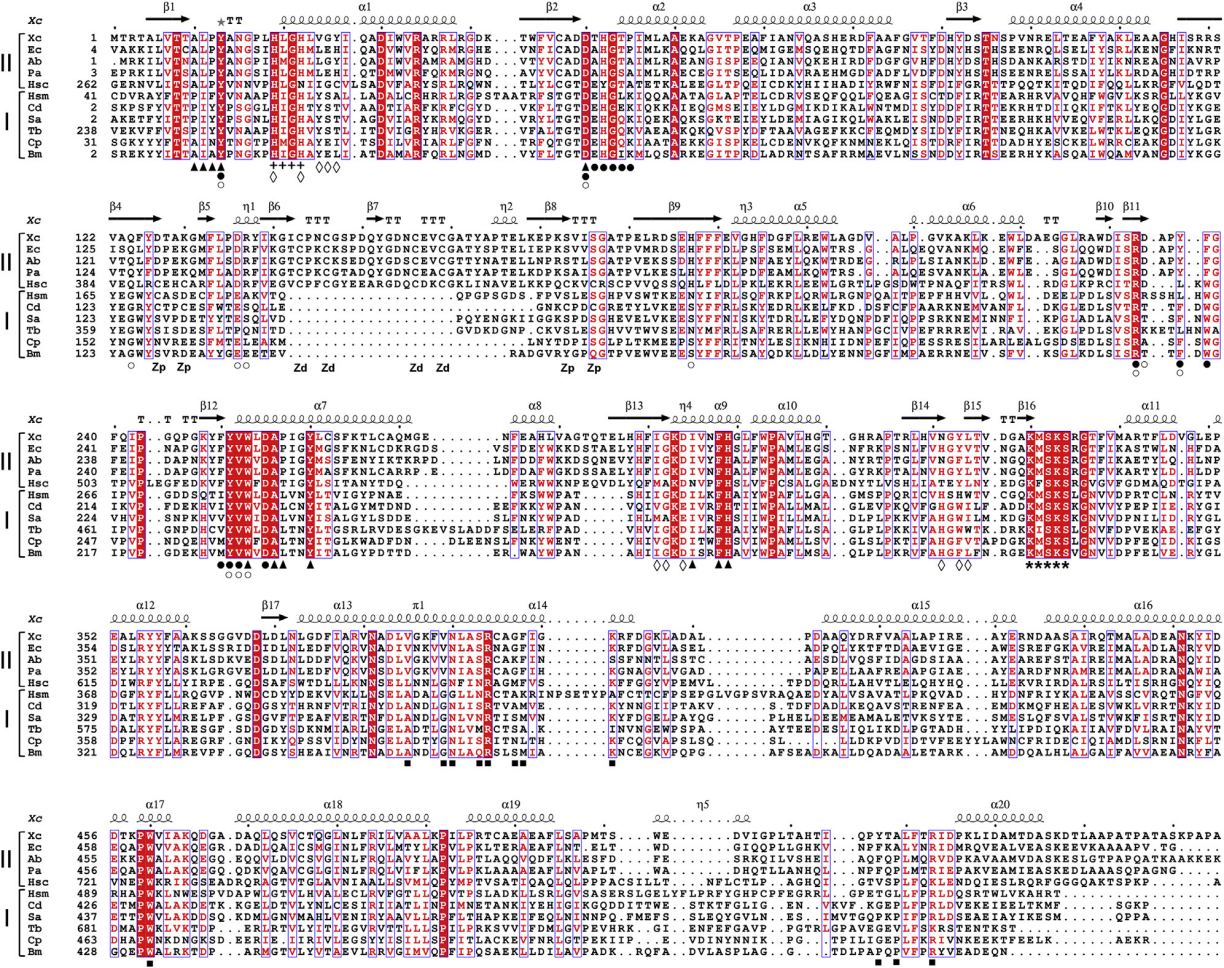
**XcMetRS is homologous to bacterial and eukaryotic type 2 MetRS.** Protein sequence alignment showing that XcMetRS (Xc, GenBank AAM36257.1) is related to several type 2 MetRS from Gram-negative bacteria (II) including *Escherichia coli* (Ec, GenBank EFJ2391260.1), *Acinetobacter baumannii* (Ab, GenBank AKQ27907.1), *Pseudomonas aeruginosa* (Pa, GenBank TEG39497.1), and the human cytosolic MetRS (Hsc, GenBank AAH02384.1). Conversely, human-mitochondrial (Hsm, GenBank AAI26295.1), *Trypanosoma brucei* (Tb, GenBank EAN77579.1), *Brucella melitensis* (Bm, GenBank EEP64467.1), *Cryptosporidium parvum* (Cp, GenBank EAK89712.1), and the Gram-positive *Clostridium difficile* (Cd, GenBank AJP13318.1), *Staphylococcus aureus* (Sa, GenBank EHS11244.1) MetRS are type 1 orthologs (I). Labels below alignments include the following: methionine pocket (▲); ATP-binding site (◊); tRNA acceptor arm site (○); anticodon-binding site (▪); auxiliary pocket (•); high motif (+); KMSKS motif (∗); and Zp and Zd, residues of the zinc-binding site in the proximal and distal knuckles, respectively. Residues highlighted in *red* and *red boxes* represent similar and conserved residues, respectively. XcMetRS secondary structure elements are indicated above the alignment. MetRS, methionyl-tRNA synthetase.

As observed for the majority of *Xanthomonas* species that infect plants, *X. citri* encodes only one MetRS, here called XcMetRS. Protein sequence analyses show that XcMetRS is a 75-kDa protein that is closely related to MetRS2 found in Gram-negative bacteria and in the cytosol of eukaryotic cells (Fig. 1). In addition to the canonical anticodon-binding domain and the classical signature motifs ‘HLGH’ and ‘KMSKS’ of class I AaRS, XcMetRS has a CP domain comprising two knuckles and a C-terminal extension that generally acts as a dimerization domain (53, 54). Moreover, the conserved zinc-binding site located in the distal CP knuckle, involving residues C143, C146, C156, and C159, suggests that XcMetRS belongs to the B family of MetRS (Fig. 1).

**Figure 2:**
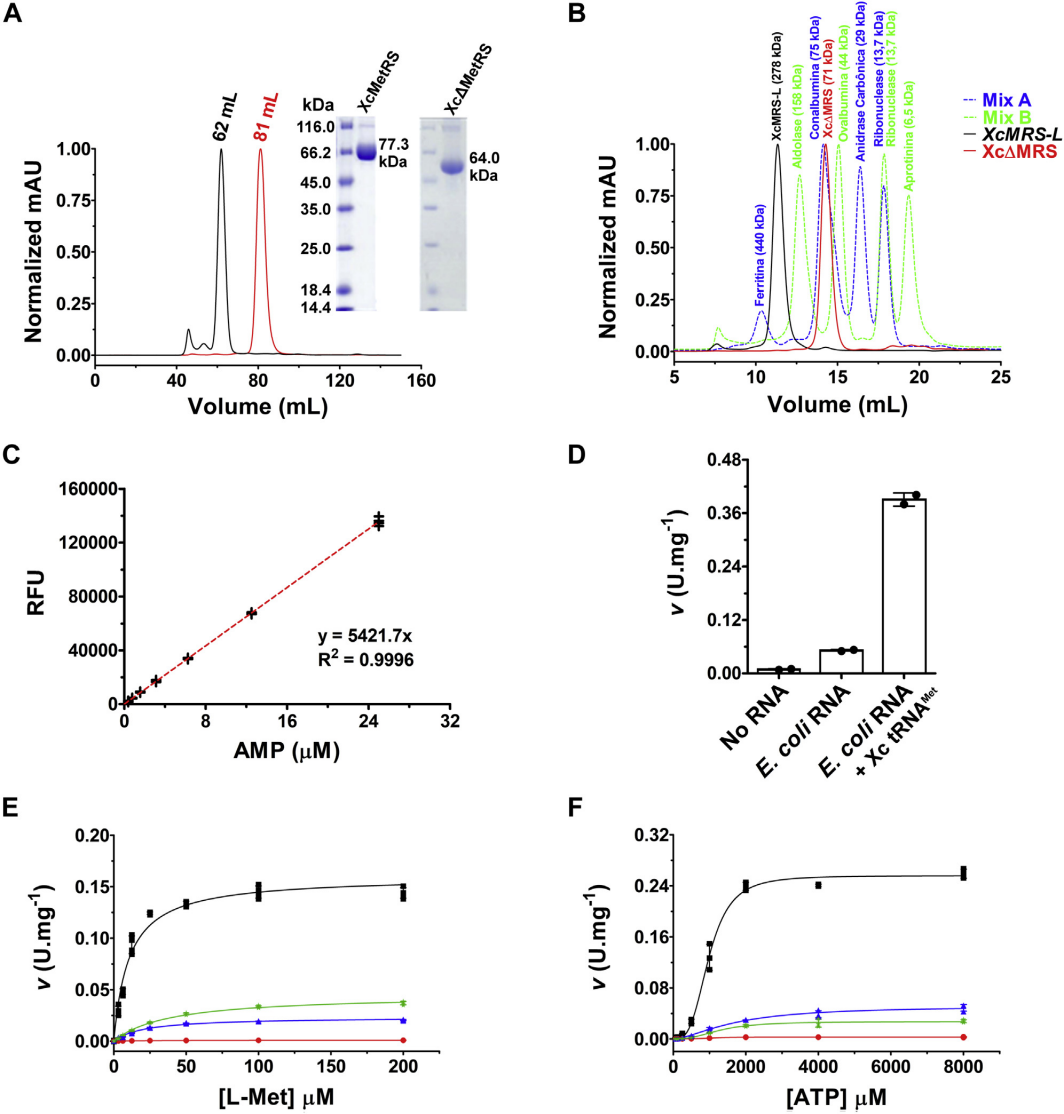
**Recombinant XcMetRS proteins form tetramers in solutions and displays aminoacylation activity upon L-Met in the presence of ATP and tRNA.***A*, a size-exclusion chromatogram showing a major peak corresponding to XcMetRS (*black line*) and XcΔMetRS (*red line*), as confirmed by SDS-PAGE (*inset*). *B*, analytical gel filtration showing that full-length XcMetRS (*black line*) elutes with an estimated molecular weight of ∼280 kDa, indicating that it is predominantly a tetramer in solution. XcΔMetRs (*red line*) eluted with a molecular mass consistent with a monomer. The molecular weight standards used to estimate the molecular masses of the sample proteins are shown in *blue* (mix A) and *green* (mix B). *C*, calibration curve showing the correlation between AMP production and the increase in relative fluorescence units (RFU) due to resazurin oxidation. *D*, activity of XcMetRS measured in the absence (no RNA) or presence of bulk *Escherichia coli* RNA from cells lacking the *Xanthomonas citri* tRNA^Met^ construct (*E. coli* RNA) or expressing the *X. citri* tRNA^Met^ (*E. coli* RNA + Xc tRNA^Met^), showing that the reaction velocity (v) is significantly enhanced when the *X. citri* tRNA^Met^ is expressed in the cells. *E*, aminoacylation activity plot of purified XcMetRS (▪) and its derivative mutants Y237L (•), P257L (▲), and Y237L/P257L (∗) upon L-Met in the presence of ATP and tRNA^Met^ showing that the mutations significantly affected enzyme activity. *F*, plot of kinetic data of XcMetRS (▪) and its derivative mutants Y237L (•), P257L (▲), and Y237L/P257L (∗) with varied ATP concentrations showing that XcMetRS has a lower affinity (*K*’) for ATP than L-Met and that the mutations also affected the affinity of the enzyme for ATP. The kinetic parameters derived from the assays depicted in panels *E* and *F* are shown in Table 1. The reaction velocities depicted in panels *D*–*F* are expressed in enzyme units (U) per mg of XcMetRS, where U corresponds to 1 μmol AMP.min^−1^ of reaction. Except for panel *D*, where two biological replicates were used, the values shown in panels *C*, *E*, and *F* correspond to measurements taken from four independent experiments or biological replicates, and error bars denote SDs. L-Met, L-methionine; XcΔMetRS, truncated version of XcMetRS lacking the C-terminal extension domain.

To test whether XcMetRS displays MetRS activity, the full-length protein was produced in *Escherichia coli* and purified by affinity and size-exclusion chromatography (Fig. 2*A*). XcMetRS eluted from the gel filtration column as a major peak with an estimated molecular mass of ∼280 kDa, indicating that it is predominantly a tetramer in solutions (Fig. 2*B*). XcMetRS activity was measured using an adaptation of a previously described method (55, 56), where the AMP produced during the tRNA aminoacylation reaction is coupled to formation of resorufin (Fig. 2*C*, Fig. S2). Notably, the activity of XcMetRS measured by the coupled assay was significantly increased in the presence of the bulk RNA extracted from *E. coli* cells expressing the *X. citri* tRNA^Met^ (Fig. 2*D*), relative to controls, indicating that the reaction is specific for tRNA^Met^ acylation.

Using this assay, XcMetRS showed a classic Michaelis–Menten curve for L-Met, with a *K*_M_^app^ of 10.5 ± 0.9 μM and a *k*_cat_ of 12.37 ± 0.28 min^−1^ (Fig. 2*E* and Table 1). A typical sigmoidal curve with positive cooperativity was obtained when ATP was used as the main substrate in concentration-dependent assays. Nevertheless, the affinity for ATP (*K*’ = 0.95 ± 0.08 mM) was significantly lower than the affinity for L-Met. In addition, the estimated *k*_cat_ and h values were 19.8 ± 0.3 min^−1^ and 3.3, respectively (Fig. 2*F* and Table 1).

**Table 1:** Kinetics data for WT XcMetRS and respective Y237L, P257L, and Y237L/P257L mutants

Catalytic parameters	WT	Y237L	P257L	Y237L/P257L
L-Met				
*K*_M_^app^ (μM)	10.5 ± 0.9	23.5 ± 0.9	21.8 ± 1.3	39.0 ± 2.0
*k*cat (min^−1^)	12.37 ± 0.28	0.07 ± 0.01	1.81 ± 0.03	3.49 ± 0.06
ATP				
*K*’ (mM)	0.95 ± 0.08	1.16 ± 0.26	2.43 ± 0.30	1.81 ± 0.29
h	3.3	4.5	1.6	2.5
*k*cat (min^−1^)	19.8 ± 0.3	0.21 ± 0.01	3.98 ± 0.22	2.03 ± 0.08
REP8839				
IC_50_ (μM)	20.8 ± 6.5	1.9 ± 0.2	>100	32.8 ± 2.7

Reported values are the means and SDs obtained from four independent measurements.

### Crystal structures of XcMetRS in its ligand-free state and bound to L-Met

To broaden our understanding of the structure–function relationship of XcMetRS, we solved the crystal structure of XcMetRS in its free form and in complex with L-Met.

Because the full-length XcMetRS did not form crystals in the initial screenings, a limited proteolysis approach was used. The trypsinized protein eluted from the gel filtration column with a much higher elution volume than the full-length XcMetRS (Fig. S3*A*), indicating a change in protein oligomeric state.

Crystals of trypsinized XcMetRS were obtained and diffracted using X-rays to a resolution of 1.7 to 2.0 Å. Using this diffraction data, the structures of XcMetRS in its ligand-free state and in complex with L-Met were solved (Table 2). In these structures, however, the electron densities for the C-terminal extension domain, known to promote protein oligomerization (53, 54, 57), were not observed.

**Table 2:** Data collection and refinement statistics of XcMetRS in ligand-free and bound states

Ligand	-	L-Met	REP8839	REP3123
Data collection statistics				
Resolution (Å)	47.83–2.00 (2.04–2.00)	47.63–1.70 (1.77–1.70)	48.96–2.00 (2.05–2.00)	42.24–1.65 (1.68–1.65)
Space group	P2_1_	P2_1_2_1_2_1_	P2_1_2_1_2_1_	P2_1_2_1_2_1_
Cell parameters				
*a*; *b*; *c* (Å)	74.0; 95.3; 84.1	77.1; 83.6; 95.2	77.2; 84.4; 95.8	76.3; 84.5; 96.3
*α* = *γ*; *β* (°)	90.0; 90.5	90.0; 90.0	90.0; 90.0	90.0; 90.0
X-rays source	W01B-MX2 (LNLS)	W01B-MX2 (LNLS)	W01B-MX2 (LNLS)	W01B-MX2 (LNLS)
Wavelength (Å)	1.4586	1.4586	1.4586	1.4586
No. of unique reflections	76,238 (4423)	68,460 (3571)	42,403 (3025)	74,214 (2797)
R_meas_^a^	0.13 (1.11)	0.08 (1.99)	0.11 (0.64)	0.08 (2.63)
Completeness (%)	97.0 (95.0)	99.9 (99.9)	98.7 (96.4)	98.3 (76.0)
<I/σ(I)>	8.6 (1.3)	19.2 (1.4)	10.2 (2.5)	12.7 (0.6)
CC_1/2_	1.00 (0.57)	1.00 (0.71)	1.00 (0.87)	1.00 (0.46)
Multiplicity	3.3 (3.1)	12.7 (12.4)	5.1 (4.8)	6.1 (4.2)
Wilson B-factor (Å^2^)	26.8	25.7	26.2	27.8
Refinement statistics				
*R*_work_	0.19 (0.26)	0.19 (0.35)	0.21 (0.32)	0.20 (0.44)
*R*_free_	0.23 (0.34)	0.23 (0.35)	0.25 (0.36)	0.23 (0.40)
Amino acid residues	1094	547	547	547
Heteroatoms (n)	2	10	28	28
Solvent (n)	498	276	155	165
Average B factor (Å^2^)				
Protein	35.2	34.5	34.1	36.1
Ligand	-	34.2	29.1	46.6
Water	39.8	37.0	35.1	38.7
Real-space correlation coefficient/Real-space R				
Ligand	-	0.94/0.10	0.97/0.11	0.96/0.11
Ramachandran				
Favored	1065 (98%)	539 (99%)	534 (98%)	539 (99%)
Allowed	25 (2%)	7 (1%)	11 (2%)	6 (1%)
PDB ID	6WQI	6WQ6	6WQS	6WQT

a R_meas_ = Σ*_hkl_* √[N/(N-1)] Σ*_i_* |*I_i_*(*hkl*) - ‹*I*(*hkl*)› | / Σ*_hkl_* Σ*_i_I_i_*(*hkl*).

**Figure 3:**
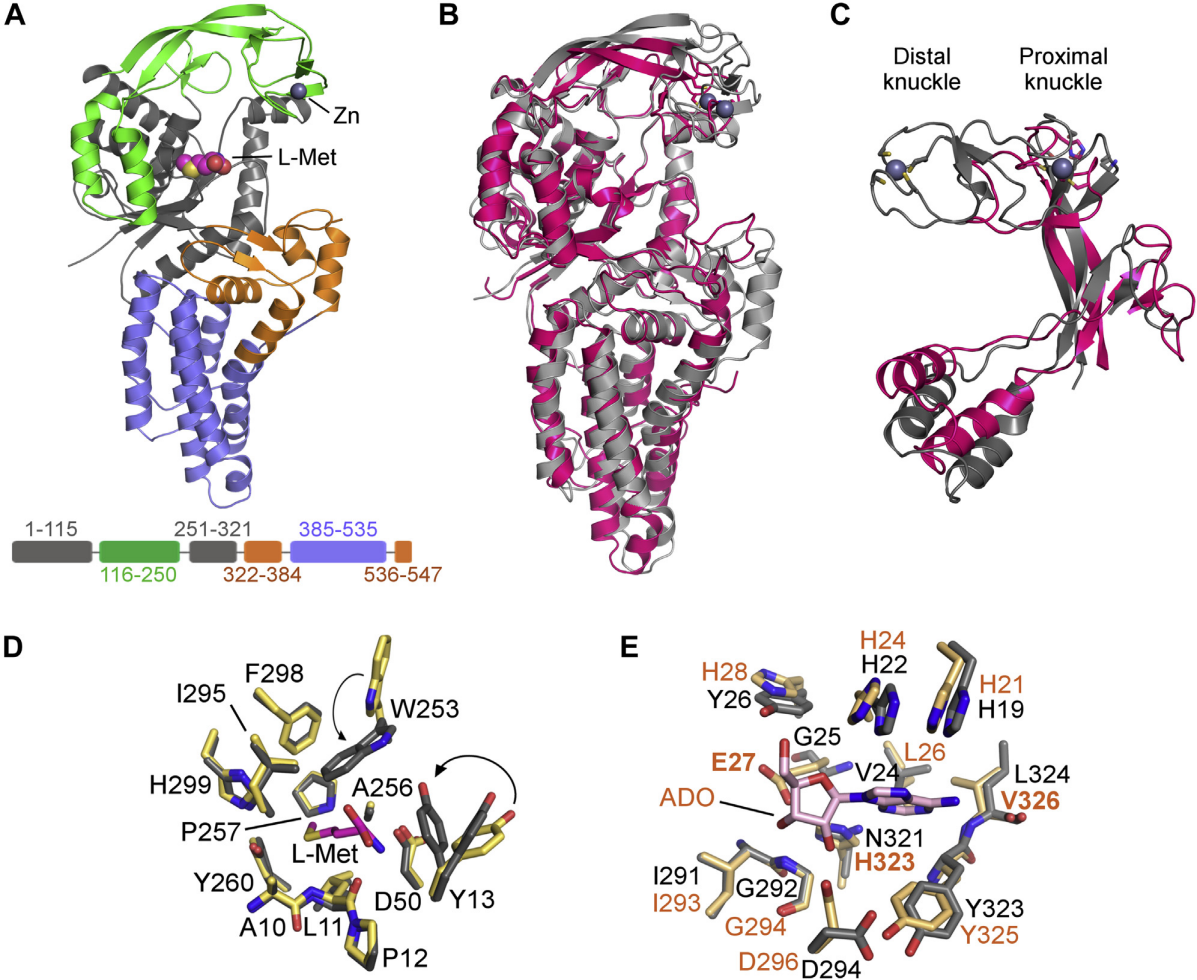
**Structure of XcMetRS and substrate-binding sites.***A*, overall structure and domain architecture of XcMetRS highlighting the classic Rossmann-fold catalytic domain (*gray*) with L-Met in the active site (*spheres*). This domain is covered by the CP domain (*green*) carrying a zinc ion (*gray sphere*) in the distal knuckle. Surrounding the catalytic domain, the KMSKS domain (*orange*) joins the anticodon-binding domain (*blue*). C-terminal extension domain (D591-R694) is not present in the structure. *B*, superposition of the crystal structures of XcMetRS (*gray*) and *Thermus thermophilus* TtMetRS (*purple*; PDB code: 1A8H) representing, respectively, type 2 and type 1 enzymes. *C*, superposition of XcMetRS (*gray*) and TtMetRS (*purple*; PDB code: 1A8H) CP domains, highlighting structural differences between type 2 and type 1 enzymes. *D*, side view representation of the XcMetRS methionine-binding site with (*gray*) or without L-Met (*light yellow*). *E*, comparison of ATP-binding pockets of MetRS from *Xanthomonas citri* and *Escherichia coli* (PDB code: 1PG2) colored in *gray* and *light orange*, respectively. The *E. coli* enzyme has an adenosine (*magenta sticks*) bound to the ATP site. CP, connective polypeptide; L-Met, L-methionine; PDB, Protein Data Bank.

XcMetRS lacking the dimerization domain has an overall structure typically found in type 1 and type 2 MetRS (Fig. 3, *A* and *B*). For instance, the alpha carbon RMSD between XcMetRS and *Thermus thermophilus* MetRS (Protein Data Bank [PDB] code: 1A8H), used as a representative MetRS1 and which is only 28% identical to XcMetRS, is 1.6 Å (Fig. 3*B*). The XcMetRS catalytic domain has a classic Rossmann fold (residues 1–115 and 251–321) comprising five parallel β-strands (β1–β3 and β13–β14) enclosed by eight α-helixes (α1–α4 and α7–α10). On top of the catalytic domain lies the CP domain (residues 116–250) that differentiates type 1 from type 2 MetRS (Fig. 3, *A* and *C*). The CP domain is made of two subdomains formed by the α5 and α6 helixes and the β4–β9 strand, respectively. The β-strand subdomain covers the active site and harbors two knuckles on its edge (Fig. 3, *A* and *C*). The distal knuckle, as predicted from the primary sequence (Fig. 1), has a zinc ion bound. Surrounding the catalytic domain, the KMSKS domain (residues 322–384 and 536–547) shows an open and poorly ordered KMSKS loop. Connected to the KMSKS domain is the C-terminal anticodon domain (residues 385–535) composed of six antiparallel (α14–α19) helixes (Fig. 3*A*).

**Figure 4:**
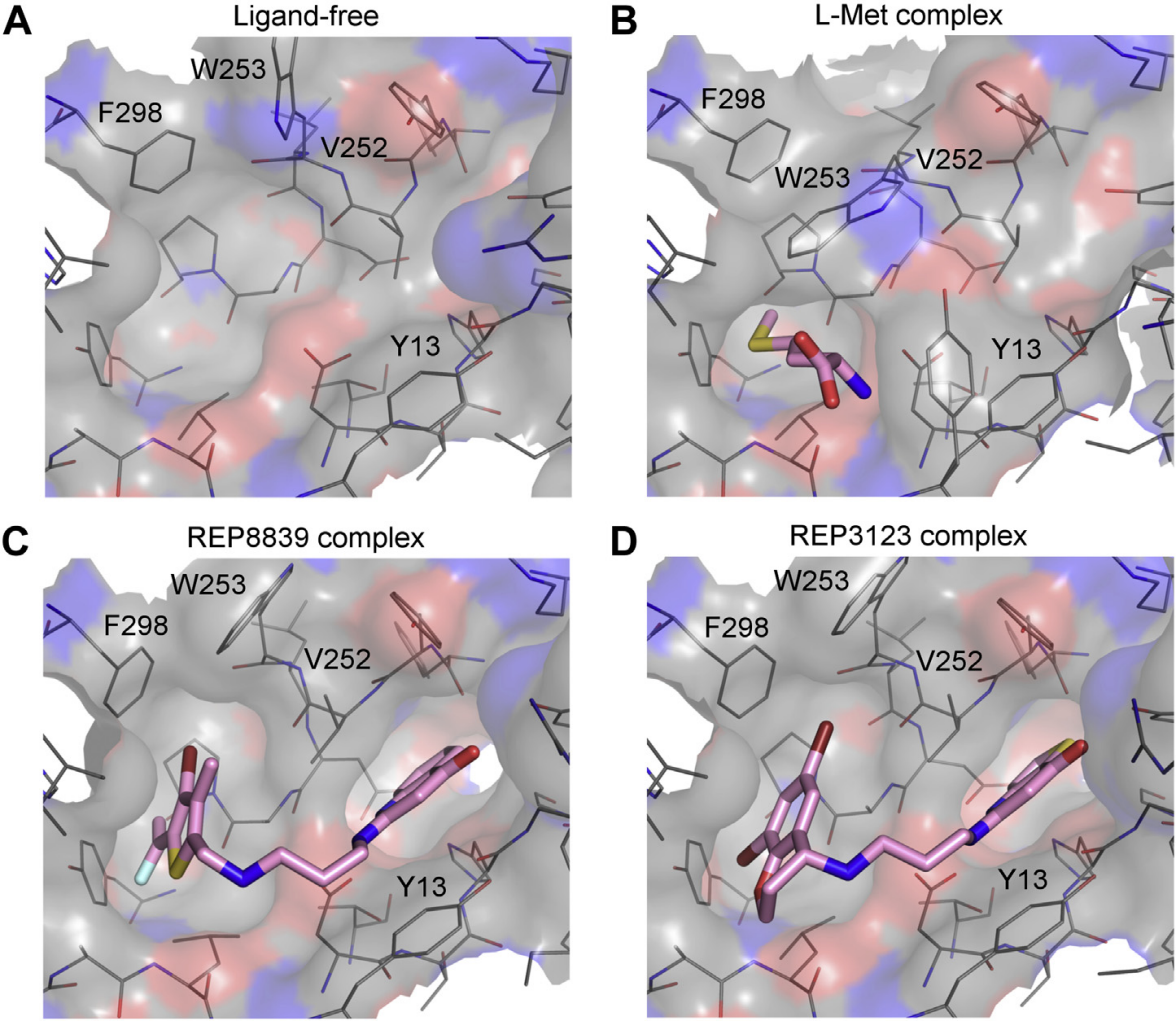
**Structures of XcMetRS in ligand-free state and bound to L-Met, REP8839, or REP3123.***A*, catalytic site without the substrate adopting an open conformation. *B*, enclosed amino acid–binding site with L-Met represented (*sticks* with *magenta*-colored carbon). *C* and *D*, complexes with REP8839 and REP3123 (*sticks* with *magenta*-colored carbon), respectively, showing that the diaryldiamines simultaneously occupy the so-called “enlarged methionine pocket” and the nearby auxiliary pocket formed upon ligand binding. Panels *A*–*D* depict the active site in the same orientation. L-Met, L-methionine.

The electron density maps of the XcMetRS structures allowed an unambiguous interpretation of the enzyme-binding pockets in the ligand-free and substrate-bound states (Fig. S4). In the ligand-free state, the methionine pocket adopts an enlarged configuration (Figs. 3*D* and 4*A*) also noticed in type 1 and type 2 MetRS from other organisms (48, 58, 59). Upon methionine binding, however, the pocket undergoes marked conformational changes involving rotamer adjustments of residues W253 and Y13 to enclose the substrate (Figs. 3*D* and 4*B*). Except for P12 and P257, the XcMetRS residues forming the methionine-binding site are highly conserved among MetRS from various organisms (Fig. 1).

XcMetRS residues involved in ATP binding, on the other hand, were mapped by comparison with the structure of the *E. coli* MetRS (EcMetRS) complexed with adenosine and methionine (27). Although the ATP-binding site is quite conserved between XcMetRS and EcMetRS, three of the 11 residues that make up this site in EcMetRS, E27, H28 and H323, are distinct from the corresponding G25, Y26, and N321 in XcMetRS, respectively (Figs. 1 and 3*E*).

### The tRNA anticodon and acceptor arm binding sites in XcMetRS

**Figure 5:**
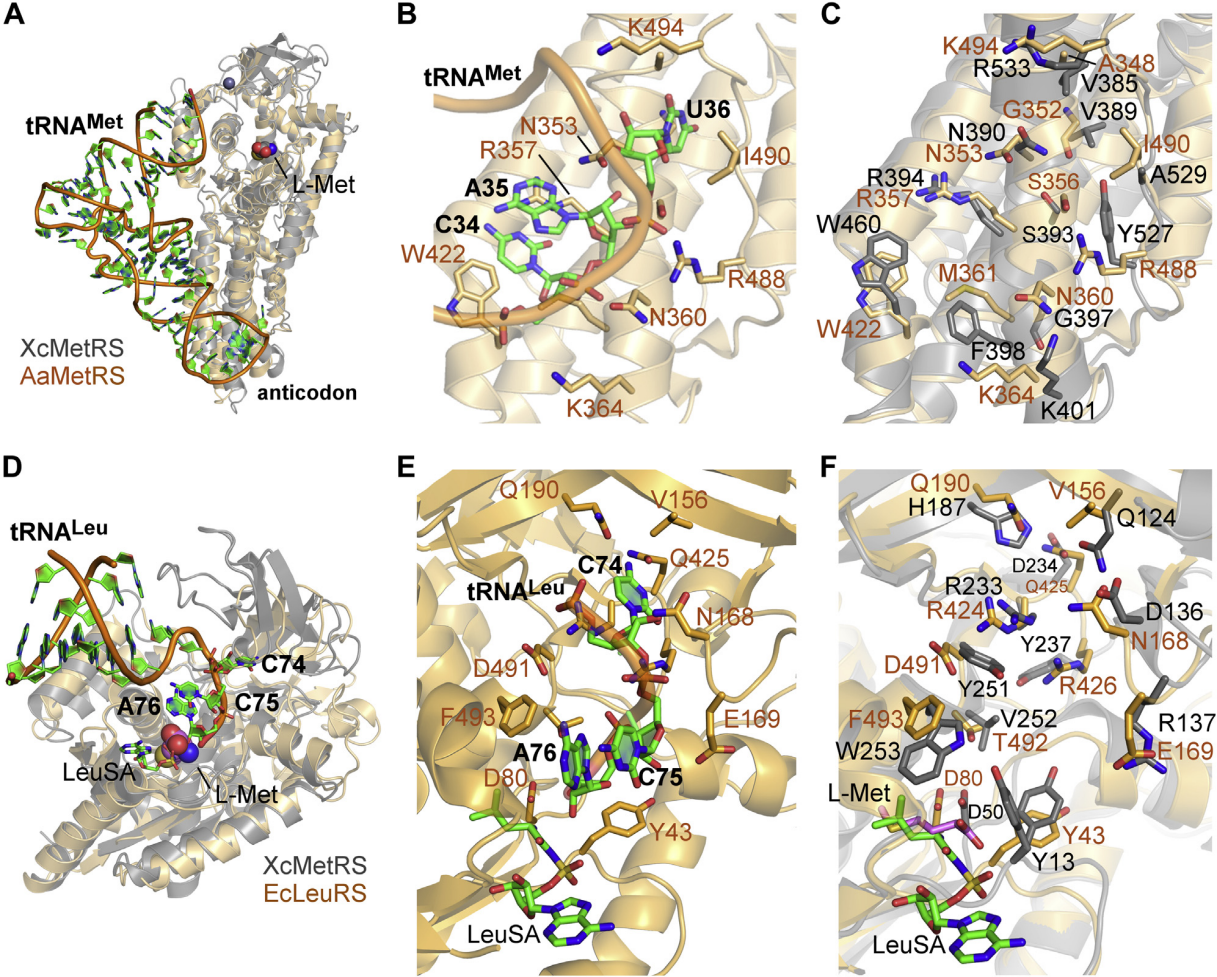
**Proposed tRNA**^**Met**^**anticodon and acceptor arm binding sites in XcMetRS.***A*, superposition of XcMetRS (*gray*) with AaMetRS (*light orange*) in complex with its cognate tRNA^Met^ (PDB code: 2CT8) showing the anticodon- and methionine-binding sites. *B*, structural detail of the anticodon recognition site in the same orientation depicted in panel *A* with the tRNA^Met^-CAU represented as *sticks* with *green* carbons. *C*, residue conservation in the tRNA anticodon recognition site between XcMetRS (*gray*) and AaMetRS (*light orange*). *D*, superposition of the catalytic domains of XcMetRS (*gray*) with EcLeuRS (*light orange*) complexed with tRNA^Leu^ and LeuSA (PDB code: 4AQ7). *E*, structural detail of the tRNA^Leu^ acceptor arm site in a similar orientation of that depicted in panel *D* with the tRNA^Leu^-CCA and LeuSA represented as *sticks* with *green* carbons. *F*, residue conservation in the tRNA acceptor arm recognition site between XcMetRS (*gray*) and EcLeuRS (*light orange*). AaMetRS, *Aquifex aeolicus* MetRS; EcLeuRS, *E. coli* Leucyl-tRNA synthetase; PDB, Protein Data Bank.

In addition to the amino acid and ATP, every AaRS can discriminate its cognate tRNA *via* two critical tRNA interaction sites, the anticodon recognition and acceptor arm binding sites (9). To date, the only known structures of a MetRS bound to its cognate tRNA^Met^ are those from *Aquifex aeolicus* (AaMetRS), PDB codes 2CSX and 2CT8 (60, 61, 62). The superposition of the XcMetRS structure with that of the AaMetRS-tRNA^Met^ complex shows that most of the residues belonging to the anticodon-binding site are conserved between XcMetRS and AaMetRS (Fig. 5, *A*–*C*). The most notable discrepancies are, nevertheless, G397, F398, and Y257 in XcMetRS, which correspond to N360, M361, and R488 in AaMetRS, respectively (Fig. 5*C*).

On the other hand, because the tRNA^Met^ acceptor arm is not visible in the AaMetRS-tRNA structures, the precise mapping of the acceptor arm binding site in XcMetRS by structural comparison to a MetRS homolog was not possible. However, because MetRS share many structural and catalytic similarities with other class I AaRS, we superimposed the structure of the catalytic domain of the *E. coli* Leucyl-tRNA synthetase (EcLeuRS, PDB code: 4AQ7) (63, 64) with that of XcMetRS in complex with L-Met (Fig. 5*D*). In the EcLeuRS structure, the tRNA^Leu^ acceptor arm adopts an aminoacylation conformation that precisely docks the adenosine 76 (A76) near the leucyl-sulfamoyl-adenylate, a reaction intermediate analogue (Fig. 5, *D* and *E*). By comparison, the XcMetRS residues potentially involved in tRNA^Met^ acceptor stem (CCA-3') recognition were mapped close to the methionine pocket (Fig. 5, *D* and *E*). This is the expected location for the acceptor arm to bind, as the tRNA^Met^ acceptor arm must adopt an aminoacylation conformation to bring the 3' terminal adenosine (commonly A76) close to the reaction intermediate (methionyl-adenylate) such that methionine can be transferred to the tRNA. In addition, the 12 XcMetRS residues predicted to form the acceptor stem binding site (Fig. 5*F*) are well conserved among MetRS from distinct organisms (Fig. 1).

### MetRS1 inhibitors bind to XcMetRS–nucleotide complexes but weakly inhibit *X. citri* growth

**Figure 6:**
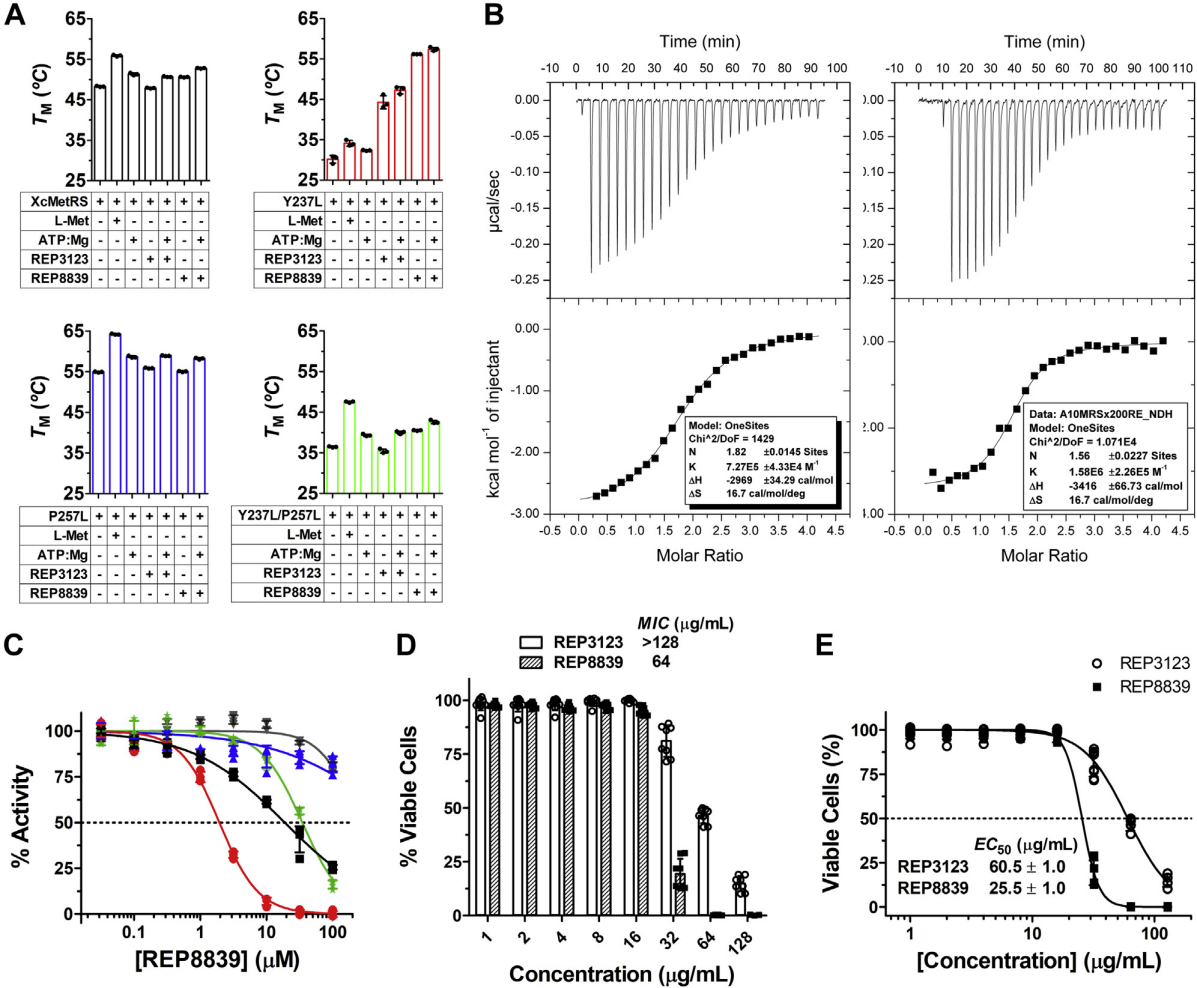
**REP8839 binds to the XcMetRS–nucleotide complex and inhibits *Xanthomonas citri* growth.***A*, DSF data showing the effect of L-Met, ATP, and the MetRS1 inhibitors REP3123 and REP8839 on the *Tm* of XcMetRS and corresponding Y237L (*red*), P257L (*blue*), and Y237L/P257L (*green*) mutants. Both L-Met and ATP significantly increased the *T*m of XcMetRS and corresponding mutants indicating that they stabilize the enzymes. A synergistic increase in the *Tm* values of XcMetRS and corresponding Y237L and Y237L/P257L mutants is observed when the diaryldiamines are combined with ATP. *B*, ITC measurements showing that REP8839 binds XcMetRS with high affinity in the presence of ATP (*left* panel) or AMP (*right panel*). Binding parameters are shown in the inset; *K*_D_ values in the presence of ATP or AMP were 1.37 ± 0.08 μM and 0.63 ± 0.09 μM, respectively. *C*, fluorescence-based enzyme assay showing the effect of REP8839 over the activity of XcMetRS (▪) and its derivative mutants Y237L (•), P257L (▲), and Y237L/P257L (∗), as well as over the coupled enzymatic reaction, as control (▼). IC_50_ values are reported in Table 1. *D* and *E*, *X. citri* growth inhibition data used to determine bacteria susceptibility to REP8839 and REP3123. Minimum inhibitory concentration (MIC) and maximal effective concentration (EC_50_) values are indicated in panels *D* and *E*, respectively. Values in panels *A*, *C*, *D*, and *E* correspond to measurements taken from three, four, eight, and four independent experiments or biological replicates, respectively, and error bars denote SDs. The mean and SD values shown in panel *A* are reported in Table S1. DSF, differential scanning fluorimetry; ITC, isothermal titration calorimetry; L-Met, L-methionine; MetRS, methionyl-tRNA synthetase; *Tm*, melting temperature.

Purified XcMetRS was analyzed by differential scanning fluorimetry (DSF) in the presence and absence of natural substrates and potential MetRS1 ligands. Without ligands, XcMetRS displayed a melting temperature (*Tm*) of 48.3 °C ± 0.1 deg. C. In the presence of L-Met or ATP, however, XcMetRS showed higher *Tm* values (55.9 °C ± 0.2 deg. C and 51.3 °C ± 0.3 deg. C, respectively) (Fig. 6*A* and Table S1), indicating that the substrates stabilize the enzyme, especially the amino acid.

In addition to L-Met and ATP, the well-known MetRS1 diaryldiamine inhibitors REP3123 and REP8839 (42, 43, 44, 46) were also evaluated by DSF. As shown in Figure 6*A* and Table S1, although REP3123 slightly reduced the XcMetRS *Tm* to 47.9 °C ± 0.1 deg. C, REP8839 increased it to 50.6 °C ± 0.1 deg. C. Interestingly, however, in the presence of ATP, REP8839 significantly shifted the enzyme *Tm* to a higher temperature by about 4.5 °C, while ATP alone shifted the *Tm* by only 3.0 °C (Fig. 6*A* and Table S1), suggesting that ATP favors the binding of REP8839 to XcMetRS.

The idea that the diaryldiamines bind more effectively to XcMetRS complexed with ATP was confirmed by isothermal titration calorimetry (ITC) assays, which showed that REP8839 binds to XcMetRS in the presence ATP or AMP with affinities of 1.4 μM and 0.6 μM, respectively, whereas in the absence of nucleotides, no *K*_D_ for REP8839 could be measured (Fig. 6*B*). On the other hand, the binding affinity of REP3123 with XcMetRS could not be obtained even in the presence of ATP or AMP because of aggregation observed during titration of this ligand (not shown).

The results shown above led us to investigate whether the diaryldiamines could inhibit XcMetRS activity. Not surprisingly, however, we found that REP8839 weakly inhibited XcMetRS activity *in vitro*, with an IC_50_ of 20.8 ± 6.5 μM (Fig. 6*C* and Table 1), a result that agrees with literature data that show that diaryldiamines, although highly potent against Gram-positive bacteria, are weak inhibitors of pathogens carrying MetRS2 enzymes, including most Gram-negative bacteria (12, 42, 43, 45). Although the inhibitory effect of REP3123 on XcMetRS activity could not be determined because of the fact that REP3123 interfered with the coupled enzymatic assay (Fig. S5), we found that REP8839 and REP3123 significantly inhibited the growth of *X. citri* at concentrations above 64 μg/ml (Fig. 6*D*). However, while REP8839 completely blocked *X. citri* growth at 64 μg/ml, with an EC_50_ of 25.5 ± 1.0 μg/ml, REP3123 showed an EC_50_ of 60.5 ± 1.0 μg/ml and still allowed *X. citri* growth at 128 μg/ml (Fig. 6*E*).

### Structures of XcMetRS in complex with diaryldiamines

To know how the diaryldiamines bind XcMetRS, relative to substrates, and why they are weak MetRS2 inhibitors, we solved the crystal structure of XcMetRS in complex with REP8839 or REP3123.

For this purpose, a truncated version of XcMetRS (residues 1–563) lacking the C-terminal extension domain (XcΔMetRS) was produced. Like trypsinized but distinct from full-length XcMetRS, XcΔMetRS eluted from the gel filtration column as a monomer in the solution (Fig. 2, *A* and *B*), further supporting that the C-terminal extension domain of XcMetRS promotes protein oligomerization. XcΔMetRS crystals were thus used to obtain the enzyme complexes with REP8839 and REP3123 (Table 2).

Again, well-defined electron-density maps of the XcMetRS structures facilitated the interpretation and modeling of the complexes with REP8839 and REP3123 (Fig. S4). The structures of XcMetRS bound to REP8839 or REP3123 revealed that these ligands occupy two distinct sites on the enzyme (Fig. 4, *C* and *D*). In the inhibitor-bound state, clear electron densities for the bromo-fluoroethenyl-methylthiophene and dibromo-chroman moieties of REP8839 and REP3123, respectively, are found in the methionine pocket, which also adopted the enlarged form observed in the ligand-free state (Fig. 4 and Fig. S4). Near the substrate binding site, however, the quinolone and thienopyrimidine moieties of REP8839 and REP3123, respectively, occupy an additional pocket, called the auxiliary pocket (Fig. 4, *C* and *D*, and Fig. S4), previously reported in protozoan MetRS1 enzymes (48). Formation of the auxiliary pocket in XcMetRS upon diaryldiamines binding requires substantial positional adjustments of the V252 and W253 residues (Fig. 4, *C* and *D*).

### Structural basis for the selectivity of diaryldiamines between type 1 and type 2 MetRS

Besides XcMetRS, the only MetRS2 structures known to date are those of *E. coli*, *Acinetobacter baumannii*, and humans (cytoplasmic). A comparison of these structures with those of MetRS1 in the free-ligand and methionine-bound states revealed that not only is the methionine pocket highly conserved in XcMetRS (Fig. 1) but also the conformational changes that occur in this pocket upon substrate binding (Figs. 3*D* and 4, *A* and *B*).

**Figure 7:**
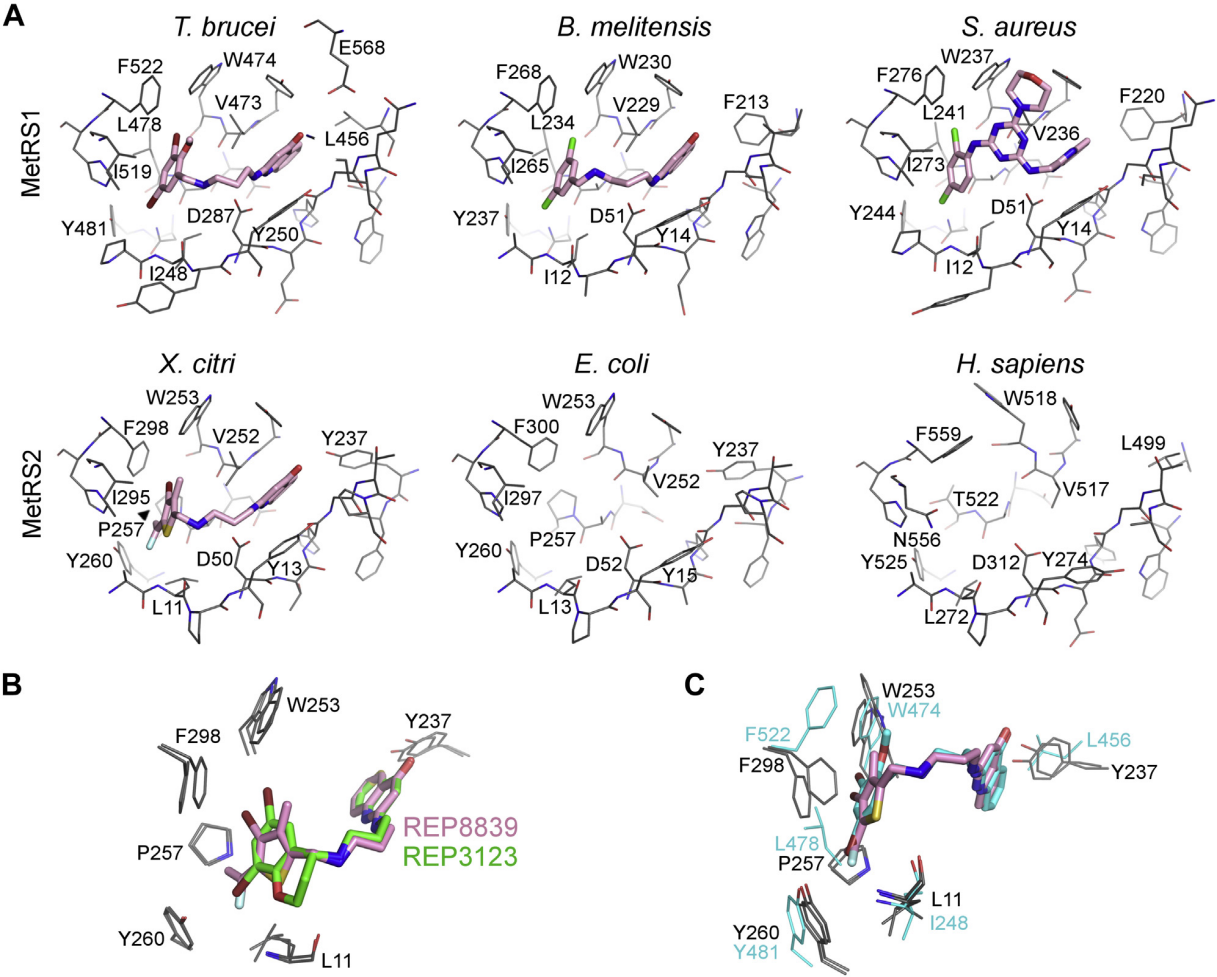
**Structural comparison between the XcMetRS structures and type 1 and 2 MetRS from bacteria and eukaryotes.***A*, XcMetRS complexed with REP8839 (*sticks* with *magenta* carbon) was chosen as a representative of type 2 MetRS in the inhibitor-bound state. The *Escherichia coli* (PDB code: 1QQT) and human (cytosolic; PDB code: 5GL7) MetRS are in free states. MetRS from *Trypanosoma brucei* (PDB code: 4EGA), *Brucella melitensis* (PDB code: 5K0S), and *Staphylococcus aureus* (PDB code: 4QRD) are shown as examples of type 1 MetRS bound to dual-site inhibitors. The methionine and auxiliary pockets on each pose are shown on the *left* and *right*, respectively. Ligands are represented as *sticks* with carbons colored *magenta*. Residues surrounding the binding pockets are show as *lines*, and those of interest are labeled. *B*, superposition of XcMetRS complexes with the diaryldiamine inhibitors. *C*, XcMetRS structures (residues represented as *lines* with *gray* carbons) in ligand-free state and bound to REP8839 (*stick* with *magenta* carbons) superposed with the *T. brucei* enzyme (carbons colored in *cyan*) bound to an inhibitor (*sticks* with *cyan* carbons). MetRS, methionyl-tRNA synthetase; PDB, Protein Data Bank.

Although XcMetRS displays virtually the same methionine-binding mode described for MetRS1 and MetRS2 enzymes, the structures of XcMetRS in complex with the diaryldiamines are the first depicting dual-site inhibitors in type 2 MetRS. This allowed us to compare the binding pockets of XcMetRS in the inhibitor-bound state with the EcMetRS2 (PDB code: 1QQT) (14, 65) and human cytosolic MetRS (PDB code: 5GL7) (66) (Fig. 7*A*). In addition, XcMetRS was compared with MetRS1 enzymes in complex with dual-site inhibitors from *Trypanosoma brucei* (PDB code: 4EGA) (48, 67), *Brucella melitensis* (PDB code: 5K0S) (35, 68), and *S. aureus* (PDB code: 4QRD) (69) (unpublished) (Fig. 7*A*).

Both the thiophene and chromane groups of REP8839 and REP3123, respectively, carry two halogen atoms. The first halogen is located close to the main-chain amide of L11 and to the hydroxyl group of Y260, whereas the second halogen approaches the side chains of W253 and F298 (Fig. 7*B*). Notably, in contrast to what is observed in type 1 MetRS, the side chain of F298 assumes a different rotamer orientation both in the ligand-free and ligand-bound structures of XcMetRS (Fig. 7*C*). In fact, the side chain of the corresponding phenylalanines in MetRS1 enzymes, including F522 in *T. brucei*, F268 in *B. melitensis*, and F276 in *S. aureus* MetRS, cannot assume another rotamer orientation because of a leucine residue that is conserved only in MetRS1 enzymes (Figs. 1 and 7, *A* and *C*). On the other hand, in XcMetRS and other MetRS2, a proline residue (P257) allows the side chain of F298 to assume an opposite rotamer orientation (Fig. 7, *A* and *C*), suggesting that distinct rotamers of F298, or equivalent residues, could affect the binding of diaryldiamines in MetRS2 enzymes.

As for the XcMetRS auxiliary pocket, one distinct feature is the presence of a tyrosine residue (Y237) that is conserved in MetRS2 from Gram-negative bacteria but which is replaced by a leucine/phenylalanine in the human cytosolic and type 1 MetRS enzymes (Fig. 7, *A* and *C*). Importantly, the hydroxyl group of Y237, which approaches the hydrophobic moieties of the diaryldiamines, is thought to weaken the hydrophobic complementarity of the auxiliary pocket with these class of inhibitors (Fig. 7, *A* and *C*).

### Polymorphic residues of the methionine and auxiliary pockets impact inhibitor binding and enzyme function

The structural comparison of the methionine and auxiliary pockets between MetRS1 and MetRS2 enzymes (Fig. 7) suggested that Y237 and P257 could contribute to the low binding affinity of diaryldiamines to XcMetRS and possibly to all MetRS2 enzymes. To test this hypothesis, the XcMetRS Y237 and P257 residues were exchanged for a leucine and a double Y237L/P257L XcMetRS mutant was also produced. The correspondent mutant proteins were purified by affinity and size-exclusion chromatography (Fig. S3*B*) and tested in the enzymatic and DSF assays.

Although the P257L mutant showed binding affinities for L-Met and ATP were only 2-fold lower than the WT XcMetRS, this mutation reduced the enzyme catalytic rates by approximately 5-fold (Table 1 and Fig. 2, *E* and *F*). However, the P257L mutant became insensitive to REP8839 inhibition (Table 1 and Fig. 6*C*), a result that is in line with the fact that REP8839 substantially altered the *Tm* of the WT but not the P257L mutant enzyme (Fig. 6*A* and Table S1).

The affinity of XcMetRS for L-Met and ATP was slightly affected by the Y237L mutation, but the catalytic rates of this mutant protein were severely impacted relative to the parental enzyme (Table 1 and Fig. 2, *E* and *F*). As predicted, the Y237L mutant showed increased susceptibility to REP8839 inhibition, with an IC_50_ of 1.94 ± 1.04 μM, a value approximately 10-fold lower than that observed for the WT enzyme (Table 1 and Fig. 6*C*). Accordingly, although the Y237L mutation significantly reduced the protein *Tm* relative to the WT XcMetRS, DSF analyses revealed that REP3123 and REP8839 dramatically increased the Y237L mutant *Tm* by approximately 14 °C and 26 °C, respectively (Fig. 6*A* and Table S1).

Finally, the Y237L/P257L double mutant showed a *K*m for L-Met approximately 4-fold greater than that observed for the WT XcMetRS, with a K’ for ATP also ∼1.8-fold greater than that of the parental enzyme (Table 1 and Fig. 2, *E* and *F*). In terms of catalytic rates, it is interesting to note that the double mutant resembles the single P257L mutant, showing turnover numbers 4- to 9-fold smaller than the WT XcMetRS. Moreover, the double mutant was also inhibited by REP8839 but showed an IC_50_ only ∼2-fold higher than the parental enzyme (Table 1 and Fig. 6*C*). Likewise, the Y237L/P257L double mutant displayed an intermediate thermal stability behavior in the apo and ligand-bound forms, relative to the single mutants and WT XcMetRS (Fig. 6*A* and Table S1).

Together, the results show that Y237L and P257L are relevant for enzyme activity and that Y237L also contributes for the lower binding affinity to the diaryldiamines.

### Diaryldiamines block the tRNA^Met^ acceptor arm site

It is notable that Y237, whose mutation to leucine drastically reduced enzyme activity (Table 1 and Fig. 2, *E* and *F*) and led XcMetRS more susceptible to REP8839 inhibition (Fig. 6*C*), is among the residues predicted to be involved in tRNA acceptor arm recognition (Fig. 5*B*). Moreover, many of the XcMetRS residues implicated in tRNA^Met^ acceptor arm binding, including Y13, D50, Y237, and V252, also map to the auxiliary pocket of the diaryldiamines (Figs. 5*B* and 7), suggesting that the tRNA^Met^ acceptor arm binding site in XcMetRS overlaps with the auxiliary pocket.

**Figure 8:**
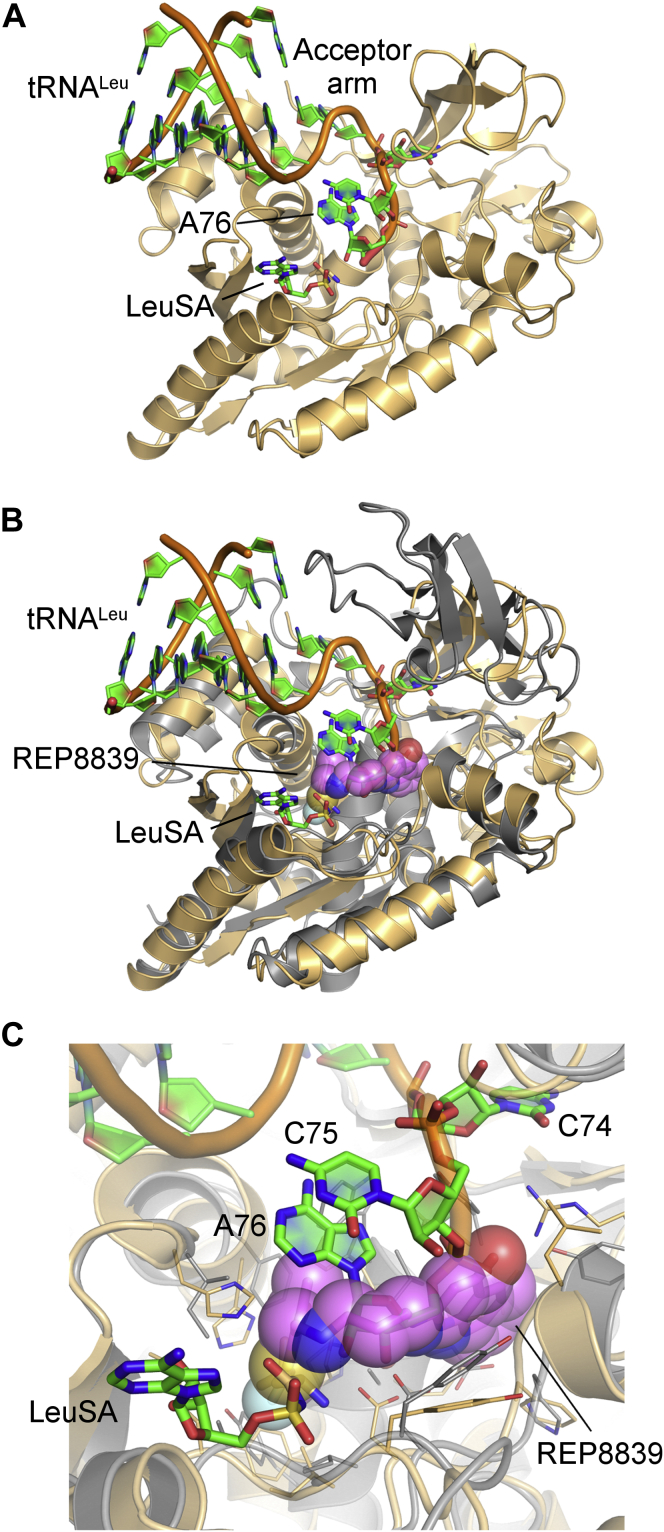
**Structural model suggesting that MetRS1 inhibitors block the tRNA acceptor arm site.***A*, catalytic domain of *Escherichia coli* LeuRS (E33-G225 and R416-M568, colored *orange*; PDB code: 4AQ7) depicting EcLeuRS with the tRNA^Leu^ acceptor arm (*sticks* with *green* carbons). The 3' terminal adenosine (A76) is positioned near the leucyl–adenylate intermediate analogue LeuSA (*sticks* with *green* carbons) in the catalytic center. *B*, superposition of the catalytic and CP domains of EcLeuRS (colored *orange*) and XcMetRS (M1-N321, colored *gray*) shows that REP8839 (*sticks* and *spheres*; carbon atoms colored *magenta*) overlaps the catalytic center of EcLeuRS. *C*, zoomed view of the catalytic center showing a detailed view of REP8839 overlap with A76 of the tRNA^Leu^ acceptor arm and the amino acid portion of LeuSA. A76, adenosine 76; CP, connective polypeptide; EcLeuRS, *E. coli* Leucyl-tRNA synthetase; LeuSA, leucyl-sulfamoyl-adenylate; MetRS, methionyl-tRNA synthetase.

To further explore this idea, we superimposed the structures of the XcMetRS-REP8839 and EcLeuRS-tRNA^Leu^ (PDB code: 4AQ7) (63, 64) complexes. We found that the diaryldiamine overlaps not only with the leucyl moiety of leucyl-sulfamoyl-adenylate located in the methionine/leucine pockets but also with A76 of the tRNA^Leu^ acceptor arm in the auxiliary pocket (Fig. 8).

Together, these observations strongly support the hypothesis that the auxiliary pocket of diaryldiamine inhibitors in XcMetRS is in fact part of the tRNA^Met^ acceptor arm binding site, which emphasizes the importance of this pocket in MetRS2 enzymes.

## Discussion

In this work, we carried out a detailed structural and biochemical characterization of the first MetRS2 enzyme from a multidrug-resistant plant bacterium belonging to the *Xanthomonas* genus, the largest group of phytobacteria that includes pathogens of great economic importance (70, 71).

XcMetRS forms tetramers in solutions in a C-terminal domain–dependent manner and has a high affinity for L-Met, but low affinity for ATP, features that are shared with many MetRS from other organisms (12, 13, 44, 53, 54, 57, 59). However, despite the similarities with MetRS previously characterized, the structures of XcMetRS presented here allowed the precise mapping of the substrates and tRNA-binding sites in this class of AaRS. The assessment of the protein structures and sequences of the XcMetRS homologs revealed that the methionine and tRNA anticodon-binding sites are the most conserved interaction sites in these enzymes. Conversely, important differences in the ATP-binding site, including residues G25 and Y26, were observed among the structurally characterized MetRS. In addition, despite being well conserved among distinct MetRS, the predicted tRNA^Met^ acceptor arm binding site of XcMetRS includes residues belonging to the CP domain (Q124, R137, and Y237) that appear to distinguish type 1 from type 2 MetRS.

This work also presents the mode of action of two diaryldiamine MetRS1 inhibitors that interact with XcMetRS, thus revealing the structural basis for selectivity of these compounds between type 1 and type 2 MetRS. Although the binding mode of the diaryldiamines to XcMetRS is reminiscent to that of type 1 MetRS, where the inhibitors also occupy the enlarged methionine and auxiliary pockets (34, 35, 36, 37, 38, 48, 58, 59), our structural data revealed amino acid differences between XcMetRS and type 1 MetRS both in the methionine and auxiliary pockets, particularly P257 and Y237, which could account for the lower binding affinity of the diaryldiamines to XcMetRS. Accordingly, the XcMetRS Y237L mutant showed increased susceptibility to REP8839 inhibition, as predicted from the structure.

Another interesting aspect of the binding mode of the diaryldiamines is the fact that the affinity of these molecules for XcMetRS is substantially increased in the presence of ATP. Because XcMetRS has low affinity for ATP, it is possible that, in addition to the amino acid polymorphisms found in the methionine and auxiliary pockets between XcMetRS and type 1 MetRS, the low ATP-binding affinity could also contribute to the lower affinity for diaryldiamines. Presumably, this class of MetRS inhibitors could effectively inhibit the growth of organisms that produce MetRS with high affinity for ATP, which is not the case of *X. citri*, as shown here.

Our mutational analysis of the main polymorphic residues found in the methionine and auxiliary pockets between XcMetRS and type 1 MetRS confirmed that such residues affect not only the binding of the diaryldiamines but also the enzyme function. This is in line with previous studies demonstrating that mutations that induce resistance to diaryldiamine inhibitors in MetRS1 enzymes also negatively affect enzyme activity (12, 42, 44).

Febrifugine and borrelidin are classical examples of inhibitors of class II prolyl- and threonyl-tRNA synthetases, respectively. Although these inhibitors also have the property to simultaneously bind multiple sites of the enzyme, including the tRNA acceptor arm site (72, 73), the observation that the auxiliary pocket occupied by the diaryldiamines in XcMetRS overlaps with the tRNA acceptor arm site had not been previously appreciated in any of the class I AaRSs. These observations thus reinforce the functional relevance of the auxiliary pocket, a druggable site that can be exploited for the development of new class I AaRS inhibitors.

Together, these results broaden our understanding of the determinants of selectivity of diaryldiamines in MetRS enzymes and provide a platform for the development of new optimized inhibitors for type 2 MetRS of Gram-negative bacterial pathogens.

## Experimental procedures

### Reagents and gene cloning

REP3123 and REP8839 were obtained from Axon MedChem. L-Met, ATP, AMP, IPTG, NAD^+^, resazurin, and trypsin (porcine pancreas) were obtained from Merck-Sigma-Aldrich. Diaphorase was acquired from Worthington Biochemical Corporation. TRIzol and SYPRO Orange were purchased from Thermo Fisher Scientific. The synthetic genes of XcMetRS (GenBank ID: AAM362577.1) and *Campylobacter jejuni* Inosine-5'-monophosphate dehydrogenase (CjIMPDH) (GenBank ID: YP_002344453.1), with codons optimized for *E. coli* expression were acquired from GenOne Biotechnologies and cloned into the BamHI and XhoI sites of the pET28–TEV vector. A truncated version of XcMetRS lacking its C-terminal domain (XcΔMetRS, M1-A563) was amplified by PCR with oligos 5'-CACCGAAAACCTGTACTTTCAAGGATCCATGAC-3' (forward) and 5'-CTCGAGTTATGCTGGCTTGCTTGCGGTTG-3' (reverse) and subcloned into the BamHI/XhoI sites of pET–TEV for bacterial expression.

The pET28–TEV plasmid carrying the synthetic *XcMetRS* gene was used as the template for site-directed mutagenesis to generate the XcMetRS mutants Y237L, P257L, and Y237L/P257L. The pairs of primers used to generate these mutants were as follows: Y237L, 5'-TGCATGGGACATCAGCCGTGACGCACCGCTGTTTGGTTTTCAGATCCCAGGCCAGCCAGGCAAGT-3' and 5'-ACTTGCCTGGCTGGCCTGGGATCTGAAAACCAAACAGCGGTGCGTCACGGCTGATGTCCCATGCA-3'; P257L, 5'-AGGCAAGTACTTTTACGTTTGGCTGGACGCACTGATTGGCTACCTGTGCAGCTTCAAAACCCTGT-3' and 5'-ACAGGGTTTTGAAGCTGCACAGGTAGCCAATCAGTGCGTCCAGCCAAACGTAAAAGTACTTGCCT-3', and the Y237L/P257L double mutant was generated with two cycles of site-directed mutagenesis.

To generate the expression plasmid carrying the *Saccharomyces cerevisiae* AMP deaminase (ScAMD) coding sequence, the *ScAMD* gene was amplified by PCR with primers 5'-GGGAATTCCATATGGACAATCAGGCTACACAGAGG-3' (forward) and 5'-ACCCTCGAGTTACTTTTCTTCAATGGTTCTCTTGAAATTGG-3' (reverse) using a cDNA library of yeast strain BY4742 as the template. The *ScAMD* fragment was cloned in pGEM-T Easy (Promega) and subcloned into the NdeI and XhoI sites of pET28–TEV.

The sequence of the *X. citri* elongator tRNA^Met^ gene (ID: tdbD00007523) was recovered from the Transfer RNA Database (74). The sequence was used to find the corresponding tRNA^Met^ in the *X. citri* 306 genome (GenBank ID: NC_003919.1). A synthetic DNA fragment corresponding to the elongator tRNA^Met^ plus 25 upstream and 40 downstream nucleotides flanking the gene of interest was obtained from FastBio and inserted into the XbaI and BlpI sites of the pET28a vector. All constructs were verified by DNA sequencing.

### Recombinant protein expression and purification

XcMetRS, XcΔMetRS, the XcMetRS mutants Y237L, P257L, and Y237L/P257L and CjIMPDH were each expressed in *E. coli* BL21(DE3), whereas ScAMD was produced in *E. coli* Rosetta 2 (DE3) cells. Cells were grown at 20 °C with 200 rpm agitation for 48 h in ZYM-5052 autoinduction media (75) containing kanamycin (100 μg/ml). After induction, cells from 0.5 l of the culture were collected by centrifugation (6000*g*, 4 °C, 30 min) and resuspended in 50 ml of buffer A (50 mM Tris HCl; 0.3 M NaCl; pH 8.0). Lysozyme (0.1 mg/ml) and DNAse I (12.5 μg/ml) were added to the cell suspensions, and after 1-h incubation at 4 °C, cells were ruptured on a Vibra-Cell VCX-500 (Sonics) sonicator. Samples were clarified by centrifugation (40,000*g*, 4 °C, 30 min) and the soluble fractions filtered on 0.45-μm Millipore filters. The recombinant proteins were purified by metal affinity chromatography using HisTrap HP columns (GE Healthcare) equilibrated with buffer A. Proteins were eluted using an imidazole gradient using buffer B (buffer A containing 0.5 M imidazole). Isolated proteins were further purified by size-exclusion chromatography on a Superdex 200 16/600 column (GE Healthcare) equilibrated with buffer C (20 mM Tris HCl; 0.3 M NaCl; pH 8.0). SDS-PAGE was used to evaluate the efficiency of the purification processes and sample purity. For molecular weight determination of full-length XcMetRS and XcΔMetRS, the purified proteins, as well as the molecular standards (mix A and B), were loaded on a 200 10/300 column (GE Healthcare) equilibrated with buffer C. The elution volumes of the molecular standards were used to estimate the molecular weight of XcMetRS and XcΔMetRS. The average distribution constant (Kav) for each protein was calculated using the formula: Kav = (Ve − V_0_)/(Vc − V_0_), were Ve is the elution volume, V_0_ is the column void volume, and Vc is the total column volume.

### RNA extraction

Based on previous reports (76), BL21 (DE3) cells were engineered to produce *X. citri* elongator tRNA^Met^. Cells carrying the pET28a_tRNA^Met^ plasmid were grown on LB medium (37 °C, 200 rpm) containing kanamycin (50 μg/ml), and tRNA expression was induced with 1 mM IPTG when culture reached an optical density of 0.6 to 0.8. After 4 h, cells were collected by centrifugation (6000*g*, 4 °C), resuspended in Milli-Q water, and subjected to RNA extraction using TRIzol. Total RNA was precipitated from the aqueous phase using isopropanol, and pellets were stored at −80 °C. Before use, RNA samples were solubilized in buffered solutions and quantified by absorbance at 260 nm using a NanoDrop 2000c reader (Thermo Fisher Scientific). Similarly, total RNA was extracted from BL21 (DE3) cells carrying no plasmid.

### XcMetRS enzyme kinetic assays

XcMetRS (E.C. 6.1.1.10) activity was measured using a continuous enzymatic fluorometric assay where AMP production was coupled to resorufin formation through the enzymes ScAMD (E.C. 3.5.4.6), CjIMPDH (E.C. 1.1.1.205), and diaphorase (E.C. 1.6.99.3). Enzymatic reactions were performed at 25 °C, with a volume of 50 μl/well in black 384-well plates, using a reaction buffer containing 0.1 M Tris HCl, pH 7.5, 0.15 M KCl, 10 mM MgCl_2_, and 0.01% Triton X-100. The L-Met *K*_M_^app^ was estimated by varying the L-Met concentration while ATP was kept at 2 mM. Likewise, the *K*_M_^app^ for ATP was determined by varying the ATP concentration while keeping L-Met at 200 μM. The assays were performed in the presence of 0.8 μg/μl of total RNA from BL21 (DE3) cells genetically engineered to expressed *X. citri* tRNA^Met^ and using 20 nM XcMetRS, 1.25 μM XcMetRS–Y237L, 160 nM XcMetRS–P257L, or 160 nM XcMetRS–Y237L/P257L. Resorufin fluorescence (λ_Exc_ 545 ± 20 nm, λ_Em_ 600 ± 20 nm) was monitored using a CLARIOstar microplate reader (BMG LABTECH). For L-Met, maximum velocity of reaction (*v*_max_^app^) and the apparent Michaelis–Menten constants (*K*_M_^app^) were obtained using the software GraphPad Prism (GraphPad Software) by adjusting the experimental data through nonlinear regression using the equation *v* = (*v*_max_^app^_x_ [S])/(*K*_M_^app^ + [S]). Similarly, *v*_max_^app^, *K*’, and Hill slope (h) were obtained for ATP using the equation *v* = (*v*_max_^app^_x_ [S]^h^)/(*K*’ + [S]^h^). The catalytic rate constant (*k*_cat_) was estimated by the equation *k*_cat_ = *v*_max_^app^/E_t_, where E_t_ is the enzyme concentration in the assay.

### Fluorescence-based enzyme inhibition assays

Inhibition studies using the XcMetRS-ScAMD-CjIMPDH-diaphorase–coupled assay (Fig. S2) were performed at various concentrations (half-log serial dilutions starting at 100 μM) of the inhibitors in anhydrous dimethyl sulfoxide (DMSO). Assays were performed at 25 °C in black 384-well microplates in 25-μl final volume in 0.1 M Tris HCl, pH 7.5, 0.15 M KCl, 10 mM MgCl_2_, and 0.01% Triton X-100. The reactions were carried out with 30 μM L-Met, 5 mM ATP, and 0.8 μg/μl of total RNA from BL21 (DE3) cells that expressed *X. citri* tRNA^Met^, and 20 nM XcMetRS, 2.0 μM XcMetRS–Y237L, 160 nM XcMetRS–P257L, or 0.5 μM XcMetRS–Y237L/P257L. Formation of resorufin was monitored in a CLARIOstar microplate reader in fluorescence mode (λ_ex_ = 545–20 nm; λ_em_ = 600–40 nm). Fluorescence signals were measured and normalized by positive (DMSO only, 100% activity) and negative (no L-Met, 0% activity) controls. Plots were prepared in GraphPad Prism.

### DSF assays

DSF was used to test the effect of L-Met, ATP, REP3123, and REP8839 over the thermal stability of XcMetRS and corresponding mutants. Assays were carried out in 96-well plates in 50 μl of buffer C containing purified XcMetRS (4 μM) and SYPRO Orange (10x) in the absence or presence of ligands. All wells contained 2% DMSO used to prepare stock solutions of REP3123 and REP8839. Protein unfolding was monitored on a 7500 Real-Time PCR System (Applied Biosystems) cycler by measuring fluorescence (λ_Emission_ = 580 nm) during 71 cycles starting at 25 °C, with a one-degree Celsius increment at each cycle. Experimental data points were normalized and adjusted to the equation *y* = *y*_*min*_ + ((*y*_*max*_ − *y*_*min*_)/(*1* + *e*^(*Tm* − *T*)/*S*^)), where *y*_*max*_ and *y*_*min*_ are normalized fluorescence values, *Tm* is the melting temperature, *T* is the temperature, and *S* is the slope.

### ITC measurements

ITC measurements were performed on a MicroCal VP-ITC calorimeter equipped with a VPViewer2000 software for data acquisition at the Spectroscopy and Calorimetry Lab facility (LEC) from the Brazilian Bioscience National Laboratory/Brazilian Centre for Research in Energy and Materials (LNBio/CNPEM). Purified XcMetRS (10 μM) was titrated against the ligands in 0.1 M Hepes, pH 7.5, containing 0.3 M NaCl, 10 mM MgCl_2_, 5% glycerol in the absence or presence of AMP or ATP at a 5 mM final concentration. To avoid nucleotide dilution, AMP/ATP was added to both ligand and receptor solutions. The ligands were added to the protein solutions kept in a 2-ml experimental cell using a titration syringe. The reactions were performed at 20 °C under agitation (200 rpm), starting with a first injection of 2 μl of ligand solution (200 μM) during 4 s, followed by 27 injections of 10 μl with 200-s intervals between injections. Titration results were corrected for ligand dilution, and all the data were analyzed using Origin7.0 software.

### Limited proteolysis

Recombinant XcMetRS purified by metal affinity chromatography was incubated with trypsin at a XcMetRS:trypsin ratio of 40:1 for 1 h at 22 °C. The sample was diluted into the binding buffer (50 mM Tris HCl, pH 8.0, 20 mM NaCl, 1 mM PMSF) and loaded onto a HiTrap Q Sepharose HP (GE Healthcare). The trypsinized protein was recovered by applying a salt gradient using the elution buffer (50 mM Tris HCl, pH 8.0, 1 M NaCl). Trypsinized XcMetRS was further purified by size-exclusion chromatography using a Superdex 200 16/600 column (GE Healthcare) equilibrated with buffer C.

### Protein crystallization and X-ray data collection

Protein crystallization experiments were carried out using the protein crystallization facility (ROBOLAB) at LNBio/CNPEM. Screenings were performed on 96-well plates using sitting-drop vapor diffusion method. A HoneyBee 963 (Digital Global) device was used to fill up the reservoirs with precipitation solutions and to mix protein samples with reservoir solutions. Sealed plates were kept at 18 °C. Initial crystals of trypsinized XcMetRS obtained with Crystal Screen HT (well A6: 30% PEG 4000; 0.2 M MgCl_2_; 0.1 M Tris HCl; pH 8.5) were reproducible in hanging-drop vapor diffusion optimizations. Suitable crystals were obtained in 10 to 15 days when 2 μl of protein solution (8 mg/ml trypsinized XcMetRS; L-Met 5 mM; AMP 2 mM; 20 mM Tris HCl; 0.3 M NaCl; pH 8.0) were mixed with 2 μl of the reservoir solution (28–32% PEG 4000; 0.2 M MgCl_2_; 0.1 M Tris HCl; pH 7.9–8.5). Crystals of trypsinized XcMetRS grown without L-Met or AMP were obtained under similar conditions but required the presence of the additive polyvinylpyrrolidone K15 (0.3 μl of 5% polyvinylpyrrolidone K15 added to a 3-μl drop made of equal amounts of protein and reservoir solution). Crystals of XcΔMetRS were obtained in hanging drops prepared by mixing 2 μl of reservoir solutions (26–30% PEG3350; 0.2 M MgCl_2_; 0.1 M Tris HCl; pH 7.9–8.5) with 2-μl protein solutions. For XcΔMetRS–REP8839 complex formation, crystals grown using a protein solution containing the substrate (8 mg/ml XcΔMetRS; 5 mM L-Met; 20 mM Tris HCl, pH 8.0; 0.3 M NaCl) were soaked for about 16 h in a cryoprotectant solution containing REP8839 (20% glycerol; 1.1 mM REP8839; 5 mM ATP; 26% PEG3350; 0.2 M MgCl_2_; 0.1 M Tris HCl; pH 8.3). On the other hand, the XcΔMetRS–REP3123 complex was obtained by cocrystallizing XcΔMetRS with the ligand (8 mg/ml XcΔMetRS; 250 μM REP3123; 20 mM ATP; 20 mM Tris HCl; 0.3 M NaCl; pH 8.0) and quick soaking of the crystals in a cryosolution containing REP3123 (20% glycerol; 10 mM REP3123; 10 mM ATP; 25% PEG3350; 0.2 M MgCl_2_; 0.1 M Tris HCl; pH 7.7). Diffraction datasets were collected at the Brazilian Synchrotron Light Laboratory (LNLS/CNPEM) beamline UVX/MX2. Typically, XcMetRS crystals diffracted to a maximum resolution of 2.6 to 1.7 Å.

### X-ray data processing, structure determination, and refinement

X-ray data were indexed and integrated using XDS (77) and scaled using AIMLESS (78) from CCP4 suite (79). The structures were solved by molecular replacement with PHASER (80), first using PDB code 1QQT (65) coordinates (EcMetRS; 48% sequence identity) (14) as the search model and afterward using XcMetRS experimental models. Real and reciprocal space refinements were performed with COOT (81) and REFMAC5 (82), respectively. MolProbity (83) was used to inspect model geometry in combination with the validation tools provided by COOT. PDB_Extract (84) was used to prepare the files for deposition into the PDB. The PDB codes for the XcMetRS structures are 6WQI, ligand-free enzyme; 6WQ6, complex with L-Met; 6WQS, complex with REP8839; 6WQT, complex with REP3123. Crystallographic statistics are presented in Table 2. Images of protein structures were prepared in PyMOL v.1.8 (Schrodinger, LLC).

### Protein sequence alignments

Protein sequences were aligned with Clustal Omega (85) and the alignment figure prepared with ESPript 3.0 (86).

### Phenotypic assays with *X. citri* 306

*X. citri* 306 was cultivated on LBON medium (10 g/l tryptone; 5 g/l yeast extract; pH 7.0) at 28 °C. The susceptibility to distinct antibiotics was evaluated using the disc-diffusion method. Briefly, a bacterial suspension was prepared in sterile saline solution (0.85% NaCl) to an optical density of 0.1 (approximately 1.5 × 10^8^ CFU/ml) and used to inoculate the surface of an LBON-agar plate (1.7% agar; 150 × 15 mm Petri dish; 55 ml/dish). Antibiotic discs were applied 15 min after inoculum, and after incubation for 24 h at 28 °C, images were recorded with a camera. The minimum inhibitory concentration was obtained through broth microdilution method. These assays were performed with eight replicates for each compound concentration. Serial 2-fold dilutions of compounds were prepared in DMSO and 1 μl transferred to sterile 96-well assay plates containing 49 μl of the culture media (LBON). Then, 50 μl of a bacterial suspension (1 × 10^6^ CFU/ml) was added to each well. Plates were incubated at 28 °C for 24 h before addition of 10 μl of resazurin 0.02%. After a new incubation (up to 4 h), the fluorescence (λ_Exc_/_Em =_ 570–615 nm) was measured using a plate reader (CLARIOstar, BMG LabTech). Blanks were prepared with culture media. Positive and negative controls were prepared with DMSO and tetracycline (10 μg/ml). The lowest concentration that causes a grown inhibition of at least 90% compared with the positive control was recorded as the minimum inhibitory concentration.

## Data availability

All data are included in the article and in the Supporting information. The atomic coordinates of the XcMetRS structures described here can be found in the Protein Data Bank under the accession codes 6WQI, 6WQ6, 6WQS, and 6WQT.

## Supporting information

This article contains supporting information.

## Conflict of interest

The authors declare no conflicts of interest with the contents of this article.
