# Supplementary material for: Molecular basis for diaryldiamine selectivity and competition with tRNA in a type 2 methionyl-tRNA synthetase from a Gram-negative bacterium
Source: J Biol Chem. 2021 Apr 12;296:100658. doi: 10.1016/j.jbc.2021.100658 (PMC8165550; doi:10.1016/j.jbc.2021.100658)
Supplement: Supplemental Figures S1–S5 and Table S1 [file mmc1.docx]

**Molecular basis for diaryldiamine selectivity and competition with tRNA in a type 2 methionyl-tRNA synthetase from a Gram-negative bacterium**

Gustavo Fernando Mercaldi^1^, Maxuel de Oliveira Andrade^1^, Jackeline de Lima Zanella^1^, Artur Torres Cordeiro^1^, Celso Eduardo Benedetti^1^

^1^Brazilian Biosciences National Laboratory (LNBio), Brazilian Centre for Research in Energy and Materials (CNPEM), CEP 13083-100, Campinas, SP, Brazil

**Supporting information**

Figure S1. *Xanthomonas citri* is a multidrug-resistant bacterium.

Figure S2. Continuous fluorescence-based AMP quantification assay for measuring XcMetRS activity.

Figure S3. Purification of trypsinized XcMetRS and XcMetRS mutants.

Figure S4. Electron-density maps of L-Met, REP8839, REP3123 and residues of the XcMetRS active site.

Figure S5. REP3123 inhibits the coupled enzyme system used to measure XcMetRS activity.

Table S1. Effect of L-Met, ATP, REP3123 and REP8839 on the *Tm* of wild type XcMetRS and corresponding Y237L, P257L and Y237L/P257L mutants.

**
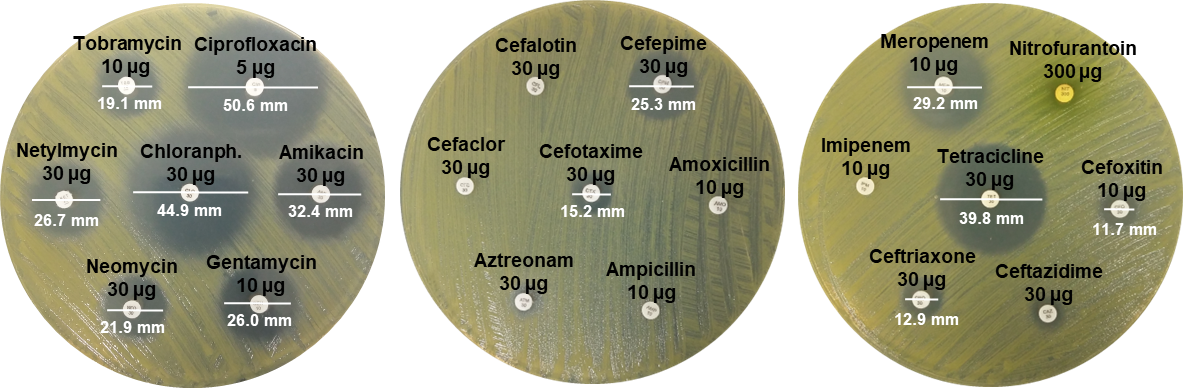
**

**Figure S1. *Xanthomonas citri* is a multidrug-resistant bacterium**. Examples of antibiograms showing that the *X. citri* strain 306 is naturally resistant to several types of antibiotics including nitrofurans (nitrofurantoin), penicillins (ampicillin and amoxicillin), monobactams (Aztreonam), cephalosporins (cephalothin, cefaclor, cefoxitin, cefotaxime, ceftriaxone, ceftazidime, cefepime) and carbapenems (imipenem). The inhibition halos formed in the presence of tobramycin and neomycin are also close to breakpoint values considered for a resistant phenotype. The amount of antibiotic is indicated on the discs.

**
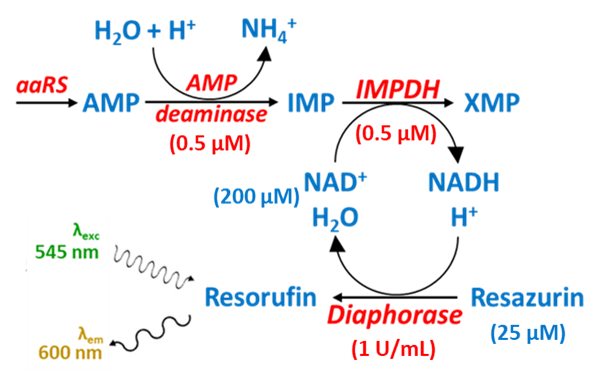
**

**Figure S2. Continuous fluorescence-based AMP quantification assay for measuring XcMetRS activity.** Scheme showing the coupled enzymatic reactions used to link AMP production to resorufin formation. AMP, adenosine monophosphate; IMP, inosine monophosphate; XMP, xanthosine monophosphate; NAD(H), nicotinamide adenine dinucleotide; IMPDH, inosine monophosphate dehydrogenase.

**
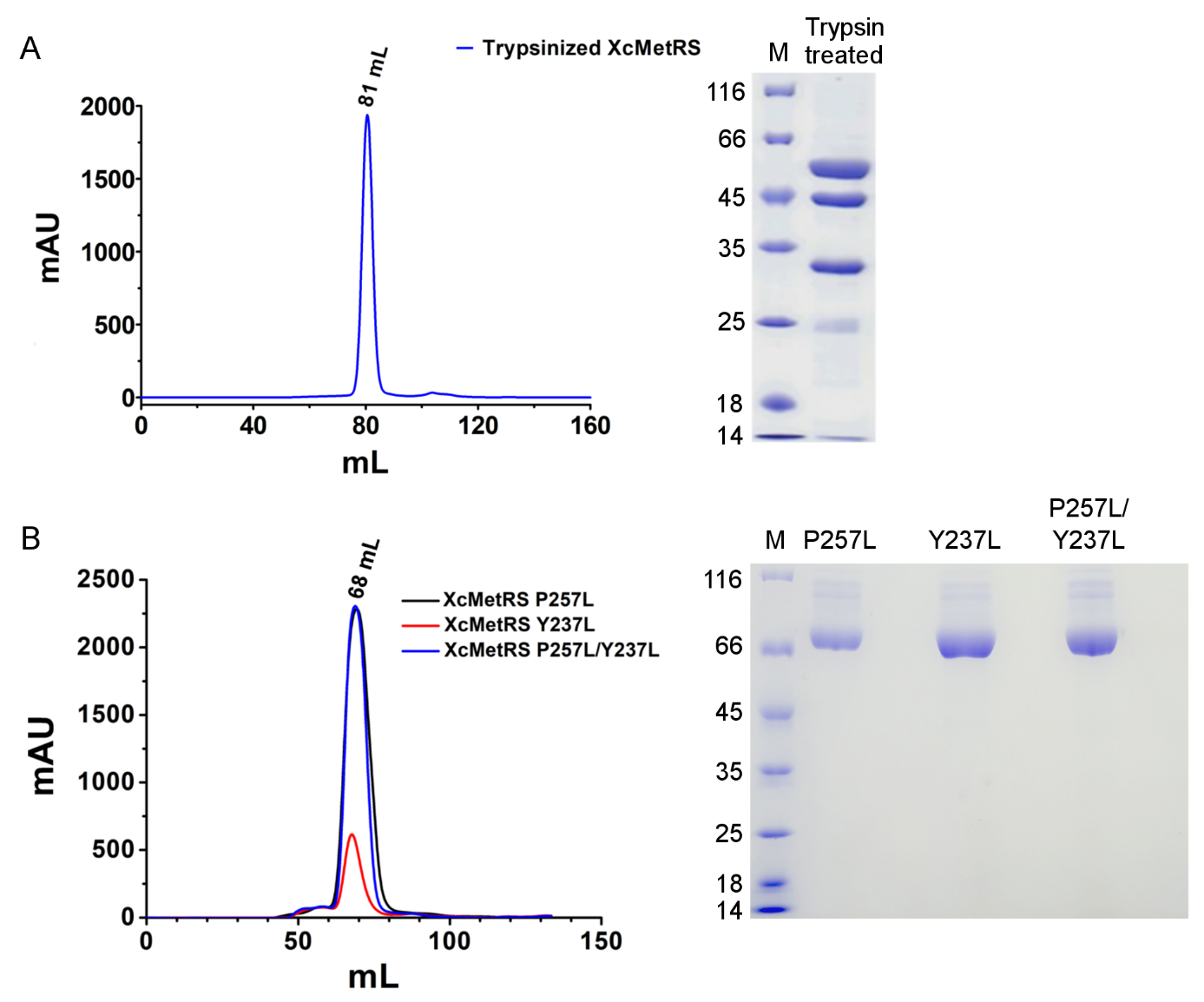
**

**Figure S3**. **Purification of trypsinized XcMetRS and XcMetRS mutants.** *A*, size exclusion chromatogram showing a major peak corresponding to trypsinized XcMetRS (left panel). When fractionated on an SDS-PAGE gel, this peak shows three major protein fragments (right panel) of smaller sizes than the undigested protein (trypsin-treated), which is consistent with the larger elution volume of this sample (81 mL) compared to that of the undigested protein. *B*, size exclusion chromatography plot (left panel) of the recombinant XcMetRS mutants, Y237L, P257L, and Y237L/P257L, showing elution volumes of approximately 68 mL, and the respective SDS gels (right panel) of the purified proteins. Molecular markers (M) are indicated on the left side of the gels.

**
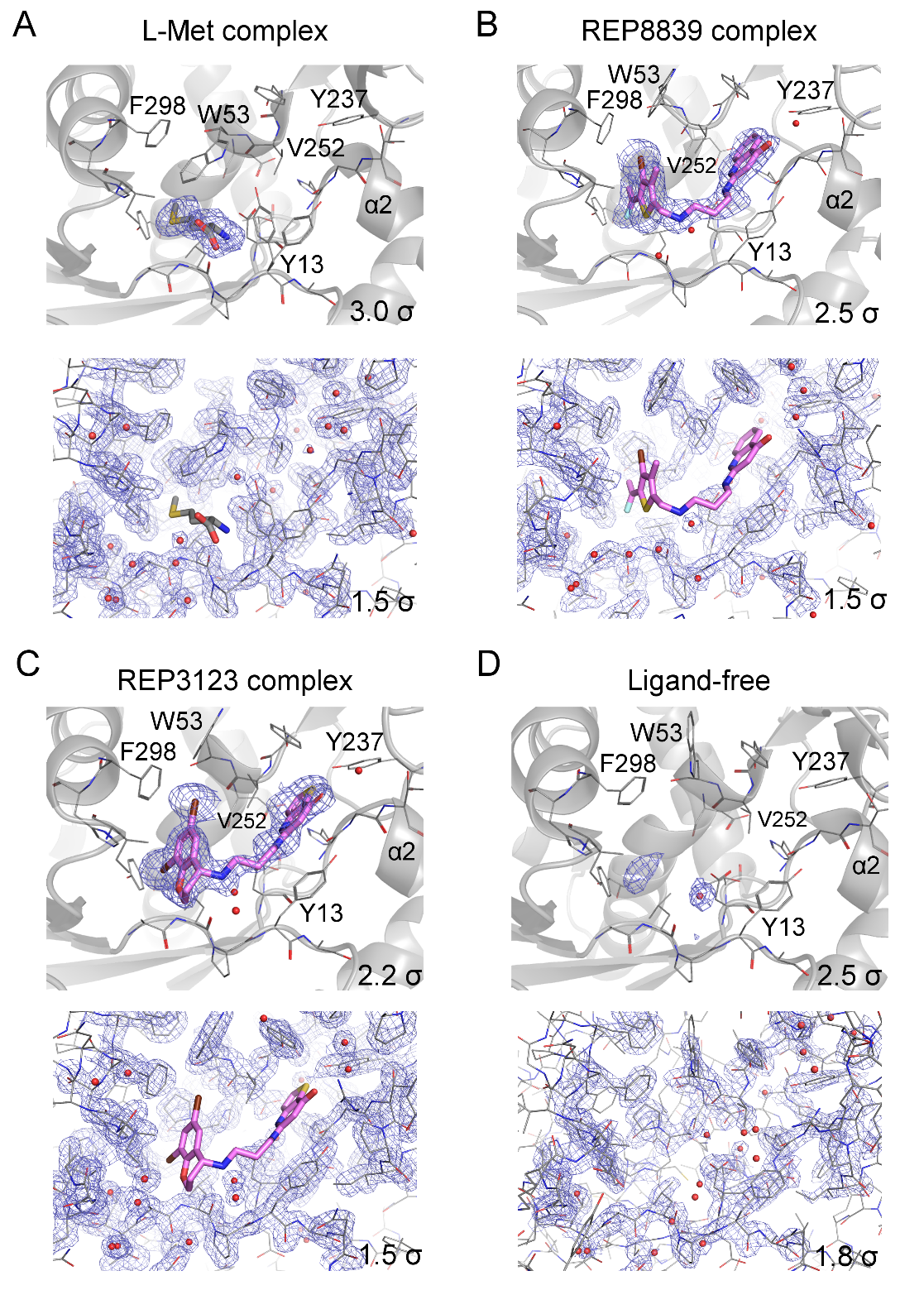
**

**Figure S4**. **Electron-density maps of L-Met, REP8839, REP3123 and residues of the XcMetRS active site.** Omit-maps (Fo-Fc) of ligands (top panels) and electron density maps (2Fo-Fc) of residues (bottom panels) obtained for ligand-bound structures of XcMetRS confirm the presence of L-Met (*A*), REP8839 (*B*), and REP3123 (*C*) in the XcMetRS active site. *D***,** by comparison, the ligand-free structure of XcMetRS has a water molecule (red sphere) in the L-Met pocket. For clarity, the electron densities of the ligands were omitted in the 2Fo-Fc maps.


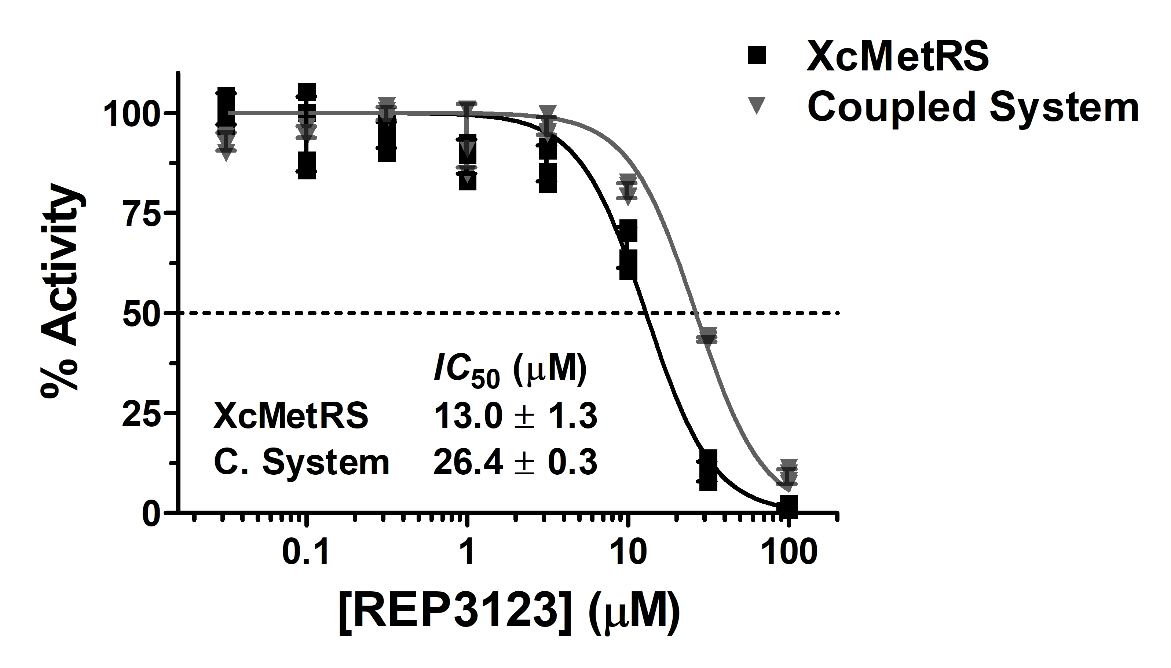


**Figure S5. REP3123 inhibits the coupled enzyme system used to measure XcMetRS activity.** Although the data suggest that REP3123 inhibits XcMetRS activity (estimated *IC*_50_ of 12.9 µM), it also significantly affected the coupled enzyme system used to detect the AMP production (*IC*_50_ of 26.4 µM) in the reaction. The interference of REP3123 in the coupled assay thus precluded the determination of a reliable *IC* _50_ value for this compound. Values correspond to measurements of four independent experiments and error bars denote standard deviations.

**Table S1. Effect of L-Met, ATP, REP3123 and REP8839 on the *Tm* of wild type XcMetRS and corresponding Y237L, P257L and Y237L/P257L mutants.**

| ***Tm* (^o^C)** | **WT** | **Y237L** | **P257L** | **Y237L/P257L** |
| --- | --- | --- | --- | --- |
| **Apo** | 48,3 ± 0,1 | 30,2 ± 1 | 55 ± 0,1 | 36,4 ± 0,2 |
| **L-Met (5 mM)** | 55,9 ± 0,2 | 34,1 ± 0,7 | 64,3 ± 0,1 | 47,4 ± 0,1 |
| **ATP:Mg (5 mM)** | 51,3 ± 0,3 | 32,3 ± 0,2 | 58,7 ± 0,3 | 39,3 ± 0,3 |
| **REP3123 25 µM** | 47,9 ± 0,1 | 44,3 ± 1,6 | 55,9 ± 0,1 | 35,4 ± 0,5 |
| **REP3123 + ATP:Mg** | 50,7 ± 0,1 | 47,3 ± 0,9 | 59 ± 0,1 | 47,4 ± 0,1 |
| **REP8839 25 µM** | 50,6 ± 0,1 | 56,1 ± 0,1 | 55,1 ± 0,1 | 39,3 ± 0,3 |
| **REP8839 + ATP:Mg** | 52,8 ± 0,1 | 57,3 ± 0,5 | 58,3 ± 0,2 | 42,6 ± 0,4 |
| Reported values represent means and standard deviations of three sample replicates. | | | | |
